# A Bystander Mechanism Explains the Specific Phenotype of a Broadly Expressed Misfolded Protein

**DOI:** 10.1371/journal.pgen.1006450

**Published:** 2016-12-07

**Authors:** Lauren Klabonski, Ji Zha, Lakshana Senthilkumar, Tali Gidalevitz

**Affiliations:** Biology Department, Drexel University, Philadelphia, Pennsylvania, United States of America; The University of Arizona, UNITED STATES

## Abstract

Misfolded proteins in transgenic models of conformational diseases interfere with proteostasis machinery and compromise the function of many structurally and functionally unrelated metastable proteins. This collateral damage to cellular proteins has been termed 'bystander' mechanism. How a single misfolded protein overwhelms the proteostasis, and how broadly-expressed mutant proteins cause cell type-selective phenotypes in disease are open questions. We tested the gain-of-function mechanism of a R37C folding mutation in an endogenous IGF-like *C*.*elegans* protein DAF-28. DAF-28(R37C) is broadly expressed, but only causes dysfunction in one specific neuron, ASI, leading to a distinct developmental phenotype. We find that this phenotype is caused by selective disruption of normal biogenesis of an unrelated endogenous protein, DAF-7/TGF-β. The combined deficiency of DAF-28 and DAF-7 biogenesis, but not of DAF-28 alone, explains the gain-of-function phenotype—deficient pro-growth signaling by the ASI neuron. Using functional, fluorescently-tagged protein, we find that, in animals with mutant DAF-28/IGF, the wild-type DAF-7/TGF-β is mislocalized to and accumulates in the proximal axon of the ASI neuron. Activation of two different branches of the unfolded protein response can modulate both the developmental phenotype and DAF-7 mislocalization in DAF-28(R37C) animals, but appear to act through divergent mechanisms. Our finding that bystander targeting of TGF-β explains the phenotype caused by a folding mutation in an IGF-like protein suggests that, in conformational diseases, bystander misfolding may specify the distinct phenotypes caused by different folding mutations.

## Author Summary

Correct protein folding and localization ensures cellular health. Dedicated proteostasis machinery assists in protein folding and protects against misfolding. Yet, folding mutations cause many conformational diseases, including neurodegenerative diseases and certain types of diabetes and cancer. Misfolded disease-related proteins interfere with proteostasis machinery, causing global misfolding in the cell. How this global mechanism leads to the specific phenotypes in different conformational diseases is unknown. Moreover, mutant misfolded proteins that only damage specific cell-types in disease often lose this cell-selectivity when overexpressed in genetic models. Here we use an endogenous folding mutation in a *C*. *elegans* secreted IGF-like protein, DAF-28, that causes dysfunction in one neuron and a specific developmental phenotype, despite expression in many cells. We find that misfolding of mutant DAF-28 causes mislocalization and defective function of another, wild-type growth factor that is expressed in the affected neuron, the TGF-β protein DAF-7. Decrease in DAF-7 function explains the observed developmental phenotype. This targeting of the bystander protein DAF-7 by the misfolded mutant DAF-28 is specific and is not caused by the global stress. Our data suggest that rather than global effects, it is the selective targeting of specific susceptible bystander proteins that defines the specific phenotypes in conformational diseases.

## Introduction

Cellular and organismal functions depend critically on the correct folding and intracellular targeting of proteins, and folding mutations are associated with many human pathologies, including neurodegenerative diseases and some forms of diabetes and cancer [1]. In addition to directly impairing the function of the affected protein, folding mutations can exhibit toxic-gain-of-function properties [2]. The mechanistic understanding of what links a specific toxic-gain-of-function mutation to the resulting disease phenotype is still very limited. One of the proposed mechanisms is global disruption of cellular folding environment, initiated by titration of chaperones, degradation machinery, and other proteostasis components by the disease-associated proteins [3–7]. We have previously shown that ectopic expression of disease proteins in *C*. *elegans* causes misfolding and loss of function of unrelated chaperone-dependent or metastable proteins in the same cell, which, in turn, drives the toxic phenotypes [5,8]. This collateral damage to normal cellular proteins by a gain-of-function mutant protein has been termed 'bystander' mechanism [9,10] and has also been shown in response to the high amyloid burden in Alzheimer's disease model as well as in other disease models [11–13]. How a single misfolded protein overwhelms the proteostasis and which cellular proteins are subject to this bystander effect are open questions. The latter question is particularly important for understanding how a broadly- or ubiquitously-expressed mutant protein can cause cell-specific dysfunction in disease. Finally, in many models of disease, mutant proteins are ectopically (over)expressed. Because such proteins may engage the proteostasis machinery differently than endogenous mutant proteins, it is important to ask whether the bystander effect can contribute to gain-of-function mechanisms exerted by endogenous mutant proteins expressed in their cognate cellular environment.

These questions about the bystander effect are particularly relevant in the endoplasmic reticulum (ER), where a single misfolded protein can cause folding stress and cellular dysfunction even though many other itinerant proteins in the ER are in the process of folding and assembly and, thus, are non-native [14–16]. Here, we ask how a folding mutation in the endogenous *C*. *elegans* insulin/IGF-like protein DAF-28 [17] affects folding or maturation of other unrelated proteins in the secretory pathway and probe the molecular events underlying the cell-selective phenotypic outcome of this mutation. We use the DAF-28(R37C) mutation to test the bystander mechanism for three reasons. First, it causes folding stress in the ER, as seen by induction of the unfolded protein response (UPR) [18]. Second, IGF proteins, the mammalian counterparts of DAF-28, are strictly dependent on the molecular chaperone GRP94 for their folding and secretion [19,20], indicating a strong engagement of this family of proteins with the ER proteostasis machinery. Third, DAF-28(R37C) mutant animals exhibit a specific and quantifiable developmental phenotype called dauer diapause resulting from dysfunction of a single chemosensory neuron (ASI), despite expression of the mutant DAF-28 protein in nine different tissues [21].

**Fig 1:**
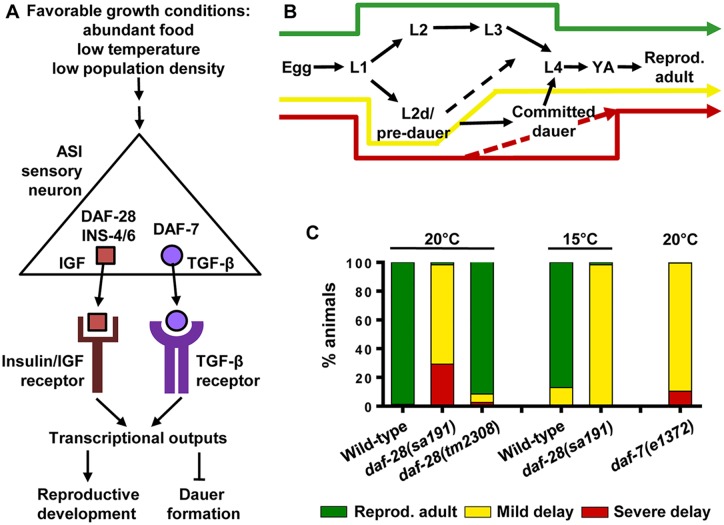
*daf-28(sa191)* gain-of-function mutation phenocopies *daf-7* loss-of-function under growth-promoting conditions. **A**. IGF-like protein DAF-28 and TGF-β protein DAF-7 are secreted from the ASI sensory neuron under growth-promoting conditions and bind to their respective receptors on target cells. This triggers transcriptional programs that promote reproductive development and prevent dauer. INS-4 and INS-6 proteins also contribute to the pro-growth insulin/IGF signaling. **B**. Possible developmental trajectories and their outcomes. Green arrow indicates normal development. Activation of dauer signaling and a brief transient entry into L2d/pre-dauer stage results in ~ 1 day developmental delay (yellow). A longer L2d duration (dashed red) or commitment to dauer development produce more severe delay (red). **C**. Percent animals in each developmental outcome for indicated genotypes. *sa191* animals activate dauer signaling even under growth-promoting conditions, as do *daf-7* loss-of-function mutants. At least three repetitions of 100–200 animals with per genotype; the raw data available in the Supplemental Table.

DAF-28(R37C) mutant protein is encoded by a semi-dominant *sa191* allele and causes inappropriate dauer entry [22]. In *C*. *elegans*, exposure of first larval stage animals (L1) to adverse conditions, such as crowding, limited food, and elevated temperature, triggers a switch from reproductive growth to an alternative stress-resistant developmental stage known as dauer diapause [23]. The decision to continue reproductive development or to enter dauer is specified by secretion of the insulin/IGF-like protein DAF-28 (referred to here as IGF-like) and the TGF-β protein DAF-7 from the ASI sensory neurons [17,24–26] (Fig 1A). The ASI neuron is the main source of the DAF-7/TGF-β in dauer signaling [27]. Loss of *daf-7* results in partial activation of the dauer state even under growth-promoting conditions, and in a complete dauer entry under sensitizing conditions, such as elevated temperature [28]. In contrast, deletion of *daf-28* does not cause dauer signaling due to redundancy with other insulin/IGF-like proteins [29,30], consistent with the gain-of-function for *sa191* allele. Overexpression of the wild-type DAF-28 or other insulin/IGF-like proteins (INS-4 or INS-6) can rescue dauer induction in *sa191* animals [17], suggesting that the mutant DAF-28(R37C) protein may have a dominant-negative effect on the wild-type pro-growth IGF-like proteins.

The R37C substitution is in a predicted RXXR proteolytic cleavage site of DAF-28 [17]. In mammalian insulins and IGFs, proteolytic processing in Golgi or secretory vesicles follows their normal folding and disulfide bond formation in the ER. Thus, the phenotype of R37C mutation may be due to misprocessing of DAF-28. However, DAF-28 with a different mutation in the same residue, R37A, does not cause a gain-of-function and is partially active [17]. Similarly, arginine substitutions in the KR cleavage site in human insulin—R89L, R89P, or R89H—result in a protein that is misprocessed but non-toxic and efficiently secreted, causing hyperproinsulinemia; however, mutation to a cysteine at the same residue—R89C—results in a severely misfolded protein and causes permanent neonatal diabetes mellitus (PNDM) [31,32]. These observations argue against misprocessing as the cause for the gain-of-function toxicity of DAF-28(R37C). Interestingly, many of the insulin folding mutations that cause PNDM and the mutant *INS*-gene-induced diabetes of youth (MIDY) generate unpaired cysteines [32,33]. Similarly, a disulfide bond-disrupting C(A7)Y mutation in the *Ins2* gene of *Akita* mouse, a diabetes model, is a toxic-gain-of-function mutation and results in insulin misfolding, induction of UPR, and eventual death of insulin-producing beta cells [34,35]. Conversely, an engineered insulin mutant carrying the *Akita* mutation but lacking all cysteines does not interfere with the wild-type insulin, despite being severely misfolded [33]. Thus, in addition to the general danger of having unpaired cysteines in the oxidizing environment of the secretory pathway, the insulin/IGF fold may be particularly sensitive to these mutations due to the topologically complex arrangement of three (four in DAF-28) disulfide bonds in a small protein.

Abnormal UPR induction is thought to be one of the mechanisms by which misfolded secretory proteins cause cellular dysfunction in many proteinopathies [36,37]. In some cases, such as in PNDM/MIDY, affected cells produce large amounts of the mutant protein, which triggers the UPR [38]. However, when the mutant protein represents only a small fraction of the non-native species in the ER, the mechanism of UPR induction is less clear, as it is not well understood what effect misfolding of one non-abundant protein has on the biogenesis of other proteins in the same compartment.

Here, we find that the R37C folding mutation in the broadly-expressed IGF-like protein DAF-28 induces defects in the protein biogenesis of the endogenous DAF-7/TGF-β expressed in the ASI neuron. The combined deficiency in DAF-28 and DAF-7 biogenesis, but not in DAF-28 alone, explains the gain-of-function phenotype of the DAF-28(R37C) mutation—deficient pro-growth signaling by the ASI neuron. This toxic effect can be modulated by ER chaperones but is observed prior to the ASI-specific UPR induction, indicating that a targeted defect in secretory protein biogenesis, rather than global ER stress, is a triggering event. Using a functional, fluorescently-tagged reporter, we find that the wild-type DAF-7 is normally localized to the sensory dendrite. However, in animals with misfolded DAF-28/IGF, DAF-7/TGF-β becomes mislocalized and accumulates in the proximal axon of the ASI neuron. The finding that bystander targeting of TGF-β explains the phenotype of a folding mutation in an IGF-like protein suggests that cellular context (*i*.*e*. the cell-specific composition of the proteome) may determine the distinct phenotypic outcomes of the different folding mutations implicated in conformational diseases.

## Results

### Gain-of-function phenotype of *daf-28(sa191)* mutation under permissive conditions

*daf-28(sa191)* mutants expressing DAF-28(R37C) protein have fully penetrant dauer arrest at elevated temperature of 25°C [22]. We have previously shown that growth at 25°C induces misfolding of temperature-sensitive and chaperone-dependent (metastable) proteins in *C*. *elegans* [5], reflecting increased burden on the proteostasis machinery. Growth at 25°C also leads to changes in stress and longevity pathways and to altered fecundity, which may, in turn, affect proteostasis [39–41]. Elevated temperature itself is highly sensitizing to dauer entry, and a further 2°C increase can cause dauer entry even in wild-type animals [42]. We tested whether *sa191* gain-of-function phenotype is still present at permissive temperatures. A majority of *sa191* animals raised under growth-promoting conditions—20°C, abundant food, and low population density—abnormally entered the pre-dauer developmental stage L2d (Fig 1B and 1C), indicating deficient pro-growth signaling by the ASI neuron (Fig 1A) [22]. As previously reported, the entry to L2d was transient, with many animals resuming the normal development within hours and some progressing to dauer before resuming the normal development. To circumvent this variability, we scored development of *sa191* animals at 20 or 15°C at 65–66 or 90–91 hours post-gastrula, respectively. At these time points, most of the wild-type animals become reproductive adults (green, Fig 1B and 1C) and any time spent in L2d and/or dauer stages is reflected as mild or severe developmental delay (yellow/red, Fig 1B and 1C). Unlike wild-type, 69±16% of *sa191* animals raised at normal growth temperature (20°C) were mildly delayed, and 29±15% were severely delayed as either early L4 larvae, dauers or pre-dauers/L2ds (Fig 1C). By contrast, only 6±1% animals with *daf-28* loss-of-function allele *tm2308* were mildly delayed and 3±4% severely delayed. At low growth temperature (15°C), 98±1% of *daf-28(sa191)* animals were still mildly delayed (Fig 1C), showing that DAF-28(R37C) mutation exerts its gain-of-function properties even at low growth temperature.

### DAF-7/TGF-β protein function is disrupted in animals with R37C mutation in DAF-28/IGF protein

**Fig 2:**
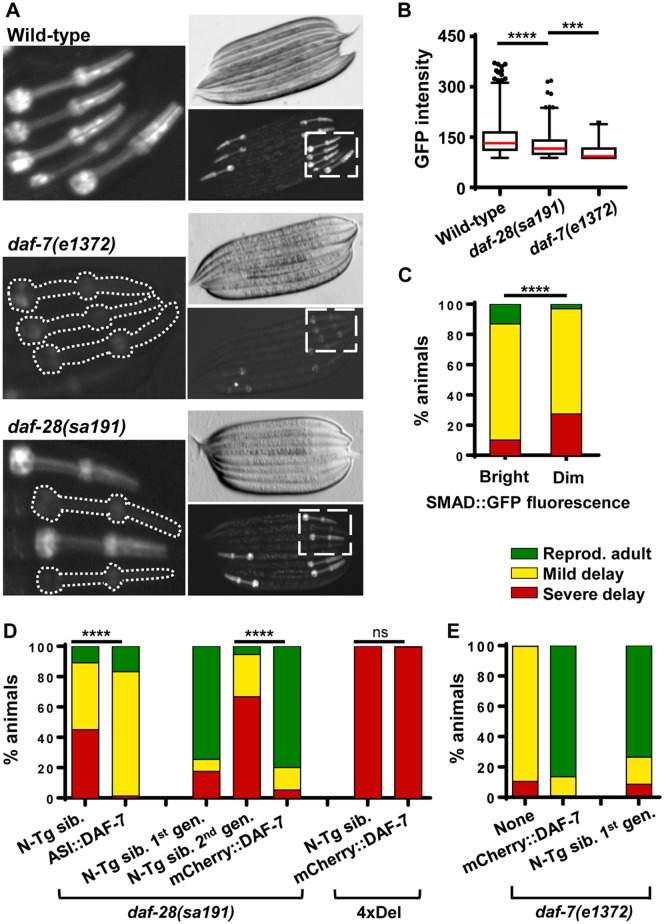
DAF-7/TGF-β protein function is disrupted in *daf-28* mutant animals. **A**. Fluorescent and transmitted light micrographs of late L1 larvae of the indicated genotypes, carrying SMAD::GFP (*cuIs5*) transgene. Bright fluorescence in the pharynx indicates DAF-7 activity. Animals were picked under transmitted light to provide an unbiased sample. Left panels correspond to boxed areas showing fluorescent pharynxes. **B**. Box-and-whisker plot of GFP intensity in animals of indicated genotypes (*n* = 1351, 770, and 118 animals, respectively; L1/L2 animals) carrying the SMAD::GFP transgene. Boxes show interquartile range, whiskers are 1-99^th^ percentile. The outliers are shown as individual data points outside the whisker range. **C**. Development of *sa191* animals with bright *vs*. dim SMAD::GFP fluorescence. Decreased reporter activity correlates with stronger activation of dauer signaling (χ^2^ = 38.81, df = 2). Animals were separated into dim and bright populations by eye using fluorescent stereo microscope. **D**. Over-expression of DAF-7 cDNA in the ASI neuron (ASI::DAF-7) (χ^2^ = 81.71, df = 2) or expression of mCherry::DAF-7 fusion protein (χ^2^ = 427.3, df = 2) rescues *sa191* dauer phenotype at 20°C. Since this is a non-integrated transgene, non-transgenic siblings (N-Tg sib.) were used as internal controls. 4xDel strain lacks four pro-growth IGF-like proteins, including DAF-28, INS-4 and INS-6. **E**. mCherry::DAF-7 fusion protein expressed from its cognate promoter rescues *daf-7(e1372)* loss-of-function mutation at 20°C. Non-transgenic siblings (N-Tg sib.) in the first generation only are also rescued. Data in B were analyzed by ANOVA followed by Bonferroni’s multiple comparison test, α = 0.05, ****P<0.0001, ***P<0.001. Data in C, D were analyzed by Chi-square test, α = 0.05, ****P<0.0001.

Entry into the L2d stage results from activation of some but not all of the converging dauer signals, such as decreased signaling from DAF-7/TGF-β [27]. DAF-7 is secreted from the ASI neurons and functions in parallel to DAF-28 (Fig 1A). Indeed, most animals carrying the *e1372* loss-of-function allele of *daf-7* entered L2d stage at 20°C, resulting in growth delay at 65–66 hours (Fig 1C). In this respect, the R37C gain-of-function mutation in DAF-28/IGF protein behaves as a phenocopy of the loss-of-function mutation of DAF-7/TGF-β. Thus, we asked whether DAF-7 protein was functional in *daf-28(sa191)* mutants. We used an established DAF-7 activity reporter—a *cuIs5* transgene expressing GFP in the pharynx from a SMAD-dependent promoter (SMAD::GFP). Reporter fluorescence is bright only when DAF-7 is secreted and is attenuated when DAF-7 secretion is low. At the late L1 larval stage, when DAF-7 and DAF-28 secretion determines the development *vs*. dauer decision, wild-type animals exhibited bright reporter GFP fluorescence (Fig 2A and 2B). In contrast, many *daf-28(sa191)* animals had decreased GFP fluorescence (Fig 2A and 2B), indicating decreased DAF-7 activity. The decrease in fluorescence was variable: some *sa191* animals were comparable to *daf-7* loss-of-function animals, while others showed intermediate to wild-type levels.

Decreased DAF-7 activity in *daf-28(sa191)* animals could due to be its transcriptional downregulation. However, *daf-7* expression does not depend on insulin signaling in *C*. *elegans*, and we did not detect a decrease in expression of its transcriptional reporter in the ASI neuron of *sa191* animals ([17] and S1A Fig).

We asked whether the variability in SMAD-dependent GFP fluorescence, the proxy for DAF-7 activity, was related to the variability in the *sa191* dauer phenotype. We separated the *daf-28(sa191);cuIs5* animals by eye into ‘bright’ and ‘dim’ populations at the end of the L1 larval stage and scored their development. Indeed, we found that *daf-28(sa191)* mutants with ‘dim’ SMAD-dependent GFP fluorescence had a higher percentage of severely delayed animals (24% v. 9%) than those with ‘bright’ fluorescence (Fig 2C).

To test whether decreased DAF-7 function contributes directly to the dauer phenotype in *daf-28(sa191)* mutants, we asked if over-expression of DAF-7 could rescue this phenotype. We used two independently generated transgenes. The first, *adEx2202*, expresses *daf-7* cDNA in the ASI neurons under the control of the *gpa-4* promoter (ASI::DAF-7) [43]. When crossed into *daf-28(sa191)* mutants, the ASI::DAF-7 transgene completely rescued the severe developmental delay, but not the mild delay (Fig 2D). Thus, the *adEx2202* transgene prevented the persistence of the L2d partial dauer stage in *sa191* animals, or their commitment to dauer, but did not completely rescue the deficient pro-growth signaling.

Second, we constructed a mCherry::DAF-7 fusion protein expressed from its cognate *daf-7* promoter, using a strategy previously used to generate functionally-tagged TGF-β proteins—*Dpp* in *Drosophila* and DBL-1 in *C*. *elegans* (S1B Fig) [44,45]. mCherry was chosen because it lacks cysteines and, thus, would not be expected to interfere with oxidative folding of DAF-7, which contains a disulfide bond-rich cysteine knot domain. The mCherry::DAF-7 protein was functional, as it efficiently rescued both characteristic phenotypes of the *daf-7(e1372)* loss-of-function allele—developmental delay/L2d entry in early larvae (Fig 2E) and the egg retention/dark intestine phenotype in adults (>99%). Surprisingly, the dauer phenotype was also rescued in non-transgenic *e1372* progeny of transgenic parents (Fig 2E, N-Tg sib.), but only in the first generation, suggesting it was due to a maternal contribution.

When expressed in *daf-28(sa191)* animals, the functional mCherry::DAF-7 protein rescued their severe developmental delay from 67% to 5±3% and supported normal reproductive development in the majority of animals (Fig 2D). Interestingly, the L2d/dauer phenotype was again rescued in the first-generation non-transgenic *sa191* progeny, indicating that the gain-of-function phenotype of DAF-28(R37C) mutation could be rescued through maternal contribution of DAF-7. Since the ASI-restricted *adEx2202* transgene did not rescue the dauer phenotype in the first-generation non-transgenic siblings (N-Tg. Sib. *v*. ASI::DAF-7, Fig 2D), the maternal rescue may depend on the non-ASI expression of DAF-7, perhaps due to the promoter or intronic elements present in our mCherry::DAF-7 transgene. Alternatively, the timing and/or strength of the *gpa-4* promoter in the *adEx2202* transgene may not be sufficient to see this rescue.

Overexpression of DAF-7 could be rescuing the dauer entry in *sa191* animals non-specifically, by simply increasing pro-growth signaling over the dauer induction threshold. If so, it should also decrease the abnormal dauer entry caused by genetic loss of all pro-growth insulin/IGF signaling. Deletion of *daf-28* alone is not sufficient to induce dauer ([29] and Fig 1C). However, a ZM7963 strain with a combined deletion of *daf-28*, *ins-4*, *ins-5*, and *ins-6* (designated as 4xDel) has a constitutive dauer phenotype at 20°C. Importantly, the dauer phenotype of this strain can be rescued by overexpression of DAF-28 alone, or by INS-4 or INS-6, due to the redundancy between deleted insulin/IGF proteins [30]. In contrast to its strong rescue of *daf-28(sa191)*, mCherry::DAF-7 had no effect on the dauer induction in the 4xDel strain (Fig 2D), indicating that it only rescues the specific defect caused by the *sa191* mutation. Taken together, our results suggest that decreased availability of secreted DAF-7 protein underlies the gain-of-function mechanism of dauer induction in animals carrying DAF-28(R37C) mutation.

### UPR caused by mutant DAF-28(R37C) is not the initiating event for ASI-specific toxicity

How could a putative folding mutation in DAF-28/IGF protein disrupt DAF-7/TGF-β activity? Since both proteins need to be secreted to signal reproductive development, and since UPR induction specifically in the ASI neuron has been implicated in the *sa191* dauer phenotype [18], we wanted to test two possible mechanisms: (1) the ASI-specific UPR, induced by the misfolded DAF-28 protein, leads to global ASI neuron dysfunction during growth *vs*. dauer decision and (2) the bystander effect, *i*.*e*. a targeted collateral damage to the endogenous cellular proteins, including DAF-7 protein, from the misfolded DAF-28(R37C).

**Fig 3:**
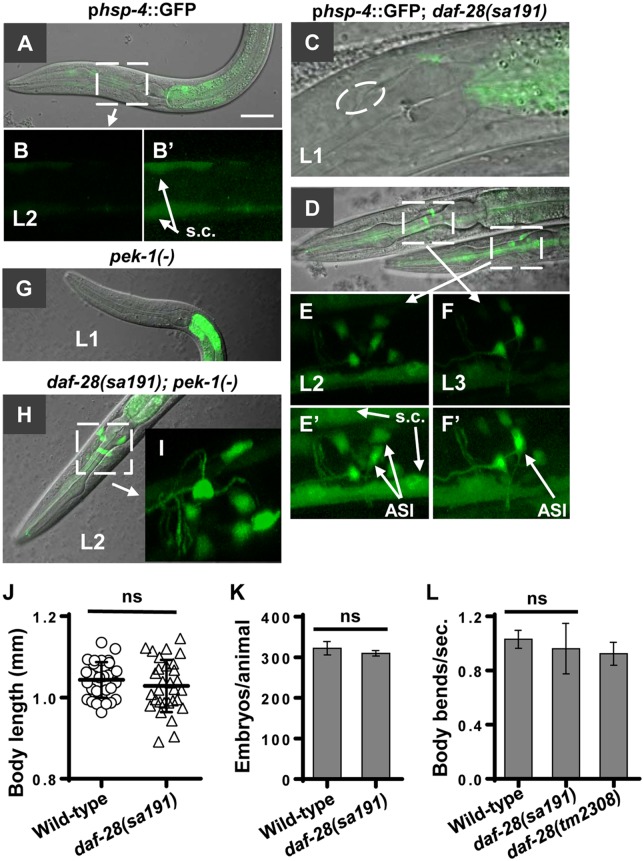
UPR caused by mutant DAF-28(R37C) is not the initiating event for ASI-specific toxicity. **A,B**. UPR reporter p*hsp-4*::GFP is not induced in neurons in wild-type animals. B’ is enhanced image of B, s.c. are seam cells. **C**. A close-up of single plane image of *sa191* L1 larva lacking UPR induction in head neurons. Oval indicates location of the ASI cell body. **D-F**. The UPR reporter fluorescence is similar in multiple cell bodies of head neurons of *sa191* L2 larvae (D,E), and is more dominant in the ASI of L3 and older *sa191* animals (D,F). E’ and F’ are enhanced images of E and F. Seam cells and the ASI cell bodies are indicated. The fluorescence in seam cells corresponds to physiological UPR during development. **G**. The UPR reporter is strongly induced in intestine of *pek-1(-)* animals (*ok275* allele). The reporter is heterozygous in all *pek-1(-)* animals. **H,I**. *pek-1* deletion does not prevent UPR reporter induction in neurons of *sa191* larvae. Fluorescent images in B,E,F and I are reconstructed z-stacks, rotated about X-axis to reveal neurons. Same settings were used to image animals in A-I. **J-L**. Expression of DAF-28(R37C) protein does not cause dysfunction in intestine or systemically (J), in reproductive tissues (K), or in ventral nerve cord (L). Data in J,K,L were analyzed by unpaired *t*-test with Welch’s correction, α = 0.05, ns = not significant.

Since overactive UPR can cause generalized cellular dysfunction and degeneration and interfere with development [46,47], we first asked if there is UPR induction in the ASI neurons of *daf-28(sa191*) animals at the time when the growth *vs*. dauer decision is made—late L1/early L2 larval stages. To visualize UPR in individual cells, we used a p*hsp-4*::GFP transgene, which has been shown to be a specific and sensitive reporter of UPR in *C*. *elegans* [48]. In wild type animals, the UPR reporter is weakly induced in the intestine and seam cells, and is undetectable in neurons (Fig 3A and 3B). Surprisingly, we did not observe a specific or strong induction of UPR in the ASI neurons of *sa191* animals at L1/L2 stages. Instead, we observed similar induction of the UPR reporter in several different head neurons of L2 animals, and no reporter induction in most L1 animals (Fig 3C, 3D and 3E). This was not due to lack of sensitivity, as we easily detected reporter expression in non-ASI cells of same animals, including seam cells and intestine (Fig 3C and 3D). Induction of the UPR reporter became more pronounced in the ASI neuron in older animals, eventually becoming the predominant source of GFP fluorescence (L3, Fig 3F).

In *C*. *elegans*, deletion of PERK increases *hsp-4* expression [49], and we observed a striking upregulation of the UPR reporter in the intestine of unstressed *pek-1(-)* animals (Fig 3G). Compared to its intensity in cells of *pek-1(-)* animals, the UPR reporter induction in the head neurons of *daf-28(sa191)* L1/L2 animals (Fig 3C–3F) was very weak. We also noted that deletion of *pek-1*, that is known to rescue dauer phenotype of *sa191* animals [18], did not eliminate ER stress in neurons of L2 *sa191* animals (Fig 3H and 3I).

The observed UPR reporter induction in intestine and seam cells of *daf-28(sa191)* animals could reflect misfolding of the DAF-28(R37C) mutant protein in these cells. In addition to the ASI and other head neurons, *daf-28* is expressed in eight other tissues, including pharynx, hypodermis, ventral nerve cord, intestine and several reproductive tissues [21]. To examine possible dysfunction of these cells, we assayed adult body size as indicator of intestinal and pharyngeal function, brood size for dysfunction in *daf-28*-expressing reproductive cells (vulva muscles, gonad sheath cells, or distal tip cell), and swimming as proxy for gross dysfunction of ventral nerve cord. We found no significant differences between wild-type animals and *daf-28(sa191)* mutants for these phenotypes (Fig 3J–3L). Previous reports also found no other deficiencies specific to the *sa191* allele beyond dauer induction [22]. Finally, as noted by Li *et*. *al*. [17], the highly transient nature of the L2d/dauer entry in *sa191* animals indicates normal function of the ASJ neuron that regulates exit from dauer, despite expression of DAF-28(R37C) protein. Thus, expression of the mutant DAF-28(R37C) protein and its activation of UPR in cells and tissues other than the ASI neuron are not sufficient to cause cellular dysfunction.

Importantly, the DAF-7 secretion defect in *sa191* animals was observed already in the late L1 stage (Fig 2A and 2B), prior to the strong and cell-specific UPR induction in the ASI neuron of older animals. Together, our data argue against the UPR being the initiating factor for the global ASI dysfunction and for the molecular events leading to the gain-of-function toxicity of DAF-28(R37C) protein.

### *sa191* gain-of-function phenotype depends on the ER protein folding environment

**Fig 4:**
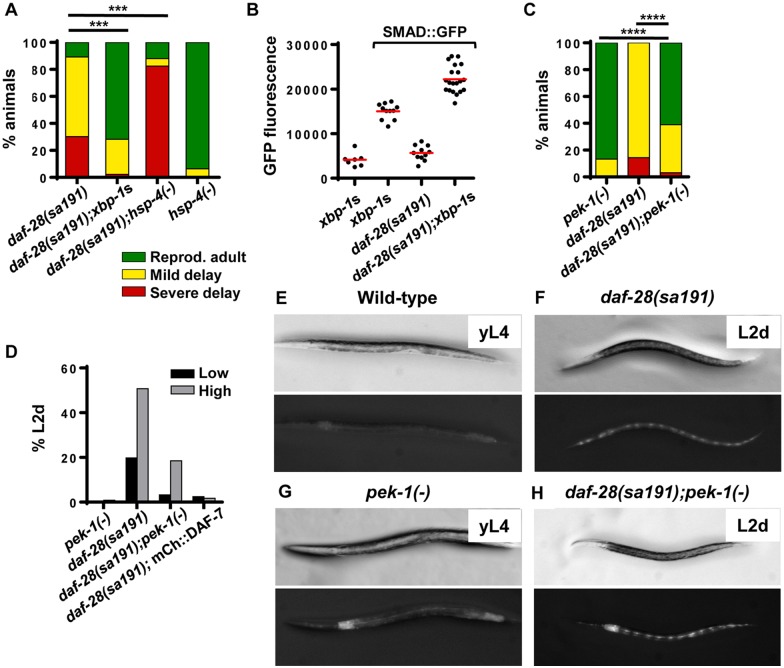
UPR signaling can modulate *sa191* gain-of-function phenotype. **A**. Pan-neuronal expression of spliced XBP-1 (*xbp-1*s) rescues the developmental delay in *sa191* animals, while deletion of HSP-4/BiP (*gk514* allele) exacerbates it (χ^2^ = 250.4 and 210.5, respectively, df = 2). **B**. Spliced XBP-1 (*xbp-1*s) rescues the decrease in SMAD::GFP fluorescence in *daf-28(sa191)* animals. Late L1 animals were imaged under YFP filter set to avoid bleed-through from the pharyngeal-expressed tdTomato used as co-injection marker for *xbp-1*s. Average fluorescence intensity of the posterior pharyngeal bulb of each animals was quantified with ImageJ. Points are individual animals; red lines indicate means. **C**. Deletion of PERK (*pek-1*(*ok275*) allele) rescues dauer phenotype in *daf-28*(*sa191*) animals at 20°C under sparse conditions (χ^2^ = 154.9, *pek-1(-) vs*. *daf-28(sa191)* and 119.3, *daf-28(sa191) vs*. *daf-28(sa191);pek-1(-)*, df = 2). **D**. Density dependence of dauer rescue by *pek-1(-)* and mCherry::DAF-7 in *sa191* animals. Low density (black) is 100–200 animals/plate; high density (gray) is 500–2,000 animals/plate. For high density, embryos were synchronized by hypochlorite treatment, plated at 500–2000 per plate, and scored for developmental delay. PERK deletion does not prevent entry into L2d/pre-dauer stage at 20°C in larger populations. **E-H**. Shown are individual animals of approximately same chronological age, taken from plates shown in S2 Fig. L2d animals are identified by partial constriction of their bodies, dark intestines, and the ‘beads-on-a-string’ fluorescence pattern of the UPR reporter p*hsp-4*::GFP, which is activated in seam cells during dauer development. Data in A and C were analyzed by Chi-square test, α = 0.05, ***P<0.001, ****P<0.0001.

If the R37C mutation indeed causes misfolding, its phenotypic expression would be expected to depend on ER chaperones and folding environment. In *C*. *elegans*, XBP-1 is a UPR transcription factor that is activated through a conserved splicing mechanism in response to the folding stress in the ER and, thus, upregulates expression of the ER chaperones and proteostasis components [48]. Importantly, transgenic expression of the active, spliced protein (XBP-1s) upregulates ER chaperone levels without causing ER stress [50]. We found that pan-neuronal expression of XBP-1s strongly rescued the gain-of-function dauer phenotype of *sa191* animals, decreasing the number of severely delayed animals from 29±18% to 3±3% (Fig 4A). This rescue, however, became less robust over several generations. This could be due to a silencing of the *xbp-1*s-expressing transgene in this genetic background, or could suggest a complex genetic interaction between the chronic folding stress and the constitutive XBP-1s activity. To ask whether the observed dauer rescue was related to improved DAF-7 activity, we measured the SMAD-GFP reporter fluorescence. Spliced XBP-1 efficiently rescued the decrease in SMAD::GFP fluorescence in *sa191* animals (Fig 4B). Of note, the positive effect of XBP-1s on the SMAD::GFP fluorescence did not attenuate over generations.

Conversely, deletion of ER chaperones would be expected to exacerbate the phenotypes caused by misfolding of DAF-28(R37C), as it decreases the cell's ability to deal with misfolded proteins. To test this, we targeted HSP-4, a stress-inducible form of the major HSP-70 ER chaperone BiP in *C*. *elegans* [48,49]. *hsp-4* is expressed in only few tissues at basal conditions, and animals with *hsp-4(gk514)* deletion appear wild-type in the absence of applied stress. Deletion of *hsp-4* resulted in increase in severely delayed animals from 29±18% to 83±6%, with majority of these being in L2d/dauer stages (Fig 4A). Together, these data show that the developmental phenotype of *sa191* allele can be modulated by molecular chaperones, consistent with the idea that misfolding of DAF-28(R37C) contributes to its gain-of-function.

Under acute ER stress conditions, activation of the PERK/eIF2α branch of the UPR allows for cellular recovery *via* transient attenuation of translation [51,52]. However, if translational attenuation was present during early development, it could lead to insufficient production of DAF-28 and DAF-7 proteins and, thus, the dauer phenotype of animals with misfolded DAF-28(R37C). Indeed, at 25°C, activation of PERK/eIF2α branch of the UPR specifically in the ASI neurons contributes to the dauer phenotype of *daf-28*(*sa191*) mutants [18]. Although we did not detect a decrease in p*daf-7*::GFP fluorescence in the ASI neurons of L1-L2 *daf-28(sa191*) larvae (S1A Fig), the GFP reporter may not be sensitive enough to detect translational attenuation. Thus, we asked whether elimination of PERK signaling was able to rescue the *sa191* phenotype under our growth conditions. Deletion of *pek-1* indeed partially rescued the gain-of-function phenotype in *daf-28(sa191)* mutants at 20°C (Fig 4C) when the animals were grown at a low density. However, we noticed that many *daf-28(sa191);pek-1(-)* animals entered the L2d stage even at permissive temperature and in the presence of abundant food when plates became crowded (Fig 4D–4H and S2 Fig). Since increased population density, and subsequent increased pheromone signaling, is one of the environmental inputs into the growth *vs*. dauer decision, loss of PERK may be selectively affecting specific aspects of the dauer signaling in *sa191* animals. Thus, we wanted to ask whether dauer rescue by deletion of *pek-1* is mediated by the rescue of DAF-7 activity, similar to what we found with spliced XBP-1. We were unable to directly assess SMAD::GFP activity due to difficulty with crosses. Instead, we asked whether rescue by DAF-7 overexpression is also sensitive to the population density and found that, unlike deletion of *pek-1*, mCherry::DAF-7 rescues the dauer phenotype of *sa191* animals even at high density (Fig 4D). Thus, rescue of the *sa191* dauer phenotype by *pek-1* deletion may not depend on increase in DAF-7 activity.

Overall, out data show that the phenotypic expression of DAF-28(R37C) mutation depends on the ER folding environment, and that the DAF-7 activity defect in these animals may be differentially affected by the two branches of the UPR.

### Expression of DAF-28(R37C) mutant protein affects axonal trafficking or local secretion

**Fig 5:**
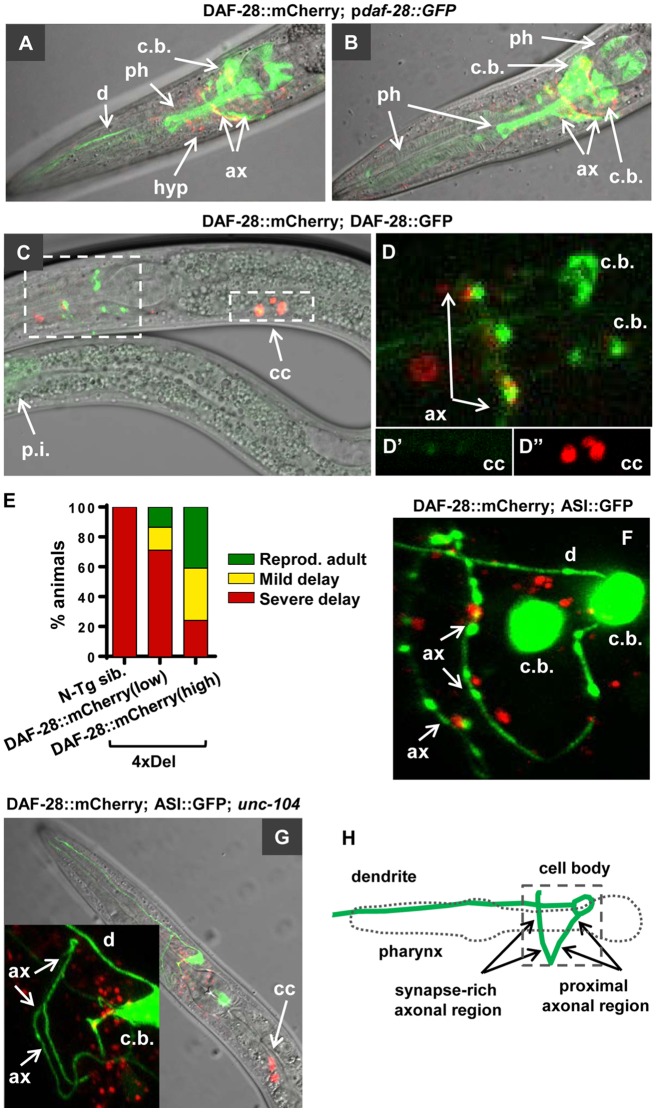
DAF-28::mCherry is expressed in punctate pattern in axons of head neurons. **A,B**. Single plane images of same animal expressing p*daf-28*::GFP transcriptional reporter (*wwEx85)* and DAF-28::mCherry protein (low expression transgene). p*daf-28*::GFP expression is seen in multiple neuronal cell bodies (c.b.), in axons (ax) and dendrites (d), in hypodermis (hyp), and pharynx (ph). **C,D**. DAF-28::mCherry protein is bright in coelomocytes (cc) of mid-L1 larva relative to its intensity in neuronal cell bodies (c.b.), indicating its efficient secretion. DAF-28::GFP accumulates in cell bodies. Both can be seen in a regular punctate pattern in axonal region. p.i. indicates posterior intestine. D, D' and D'' are enlarged areas boxed in C. D' and D'' are green and red channels of the same image. **E**. DAF-28::mCherry protein rescues persistent dauer entry of the 4xDel strain (missing four pro-growth IGF-like proteins, including DAF-28, INS-4 and INS-6). **F**. DAF-28::mCherry protein (red) localizes in a regular pattern in large puncta adjacent to the ASI axon, as well as in pharyngeal and hypodermal tissues. Green, p*daf-7*::GFP;*rol-6* array used to visualize ASI neuron (ASI::GFP). Orientation of the ASI neurons is shown in panel H. **G**. Inactivation of the *C*. *elegans* homologue of axonal kinesin KIF1A, *unc-104*, eliminated axon-adjacent puncta of DAF-28::mCherry. Accumulation of mCherry-positive vesicles, presumably representing the DAF-28::mCherry secreted from the cell body, can be seen instead. **H**. Schematic representation of orientation of the ASI neuron relative to the pharynx. Anterior is to the left, dorsal up. The ASI cell body, dendrite, and the proximal and synapse-rich distal axonal regions are indicated. All confocal images of ASI neurons follow this orientation.

Since we found that UPR does not trigger the dauer signaling in *daf-28(sa191)* animals, we tested the second proposed mechanism—bystander misfolding of endogenous cellular proteins. We considered two potential targets: other pro-growth insulin/IGF-like proteins and DAF-7. To examine the effect of mutant DAF-28(R37C) on wild-type insulin/IGF-like proteins, we generated a wild-type DAF-28::mCherry and followed its localization in wild-type and *sa191* animals. The DAF-28::mCherry expression followed the reported expression pattern of p*daf-28*::GFP transcriptional reporter, with fluorescent protein detected in head and tail neurons, pharynx, hypodermis, and other tissues (Fig 5A and 5B). DAF-28::mCherry was efficiently secreted upon expression in L1 stage, as detected by its uptake by endocytic scavenger cells, coelomocytes [53] (Fig 5C and 5D). Interestingly, compared to the DAF-28::mCherry fusion protein, the previously described DAF-28::GFP protein [17,54] was not efficiently secreted, as judged by coelomocyte fluorescence, and instead appeared to accumulate in neuronal cell bodies (Fig 5C and 5D). As discussed below, this may be due to misfolding of the GFP moiety.

To verify the functionality of the DAF-28::mCherry fusion protein, we crossed it into the 4xDel strain. Despite mosaic expression, DAF-28::mCherry protein efficiently rescued the dauer phenotype of the 4xDel strain, showing that this protein is functional (Fig 5E).

Confocal imaging showed that DAF-28::mCherry protein is predominantly found in a punctate pattern reminiscent of the secretory vesicles in mammalian cells expressing insulin or IGF, with some puncta found in a regularly spaced pattern adjacent to the ASI axons (Fig 5F). Inactivation of *unc-104*, the *C*. *elegans* homologue of the axonal kinesin KIF1A, eliminated these axon-adjacent puncta (Fig 5G), suggesting that they represent either local accumulation of DAF-28::mCherry protein secreted from the ASI axon, or protein present in axons of other neurons expressing *daf-28*. The orientation of the ASI cell body, axon and dendrite is shown in Fig 5H.

**Fig 6:**
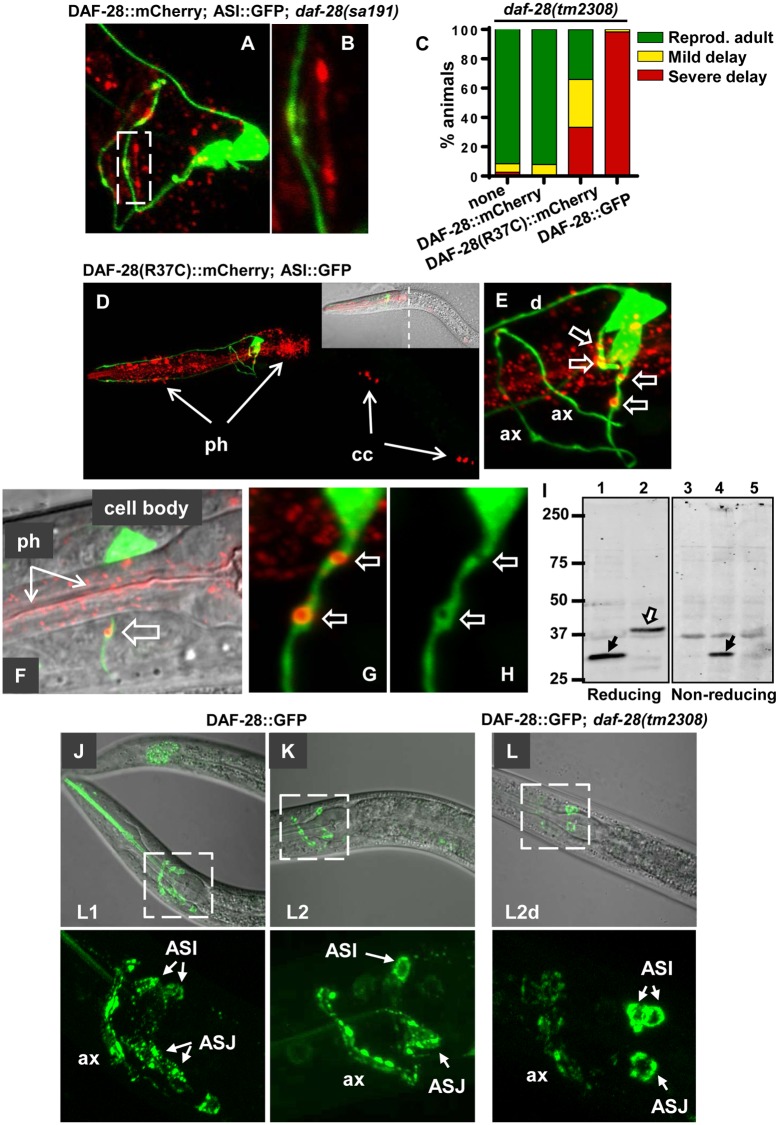
Axonal defects in animals expressing the mutant DAF-28(R37C) protein. **A,B**. The peri-axonal pattern of DAF-28::mCherry protein is irregular in *daf-28*(*sa191)* animals. B corresponds to the boxed area in A. All animals in A-H carry the p*daf-7*::GFP;*rol-6* array (*ksIs2*) to visualize ASI neurons (green). **C**. Expression of DAF-28(R37C)::mCherry or DAF-28::GFP (*svIs69*) causes gain-of-function dauer phenotype in *daf-28* deletion background. **D-H**. DAF-28(R37C)::mCherry protein accumulates in the aggregate-like structures in the proximal axon of the ASI neuron (block arrows) and disrupts its architecture. D shows a single L1 animal, E is a close-up on its ASI neurons. Note also strong pharyngeal accumulation of the DAF-28(R37C)::mCherry protein (ph, arrows), and its presence in coelomocytes (cc). F-H show aggregate-like accumulations of the mutant protein in a proximal axon. F is a single plane image, all other fluorescent images are reconstructed z-stacks. **I**. DAF-28(R37C)::mCherry protein (white arrow, lanes 2 and 5) is not resolved under non-reducing conditions. Worms with mCherry alone (black arrow, lanes 1 and 4) and non-transgenic animals (lane 3) were used as controls. **J-L**. DAF-28::GFP protein progressively accumulates in neuronal cell bodies and axons during development in wild type animals, and in L2d stage in *daf-28* deletion background. Note the transition from a punctate pattern in cell bodies to ‘filled-in’ appearance. ax indicates axons; ASI and ASJ indicate their cell bodies.

Misfolded insulin with unpaired cysteines can exert a dominant-negative effect on the wild-type insulin [33,55]. To determine whether interference with biogenesis of wild-type insulin/IGF-like proteins contributes to the gain-of-function of *sa191* allele, we crossed *sa191* animals to those expressing the functional DAF-28::mCherry protein. DAF-28::mCherry was still secreted, and we did not detect major redistribution of the fluorescent signal to the cell bodies as would be expected if the protein was retained in the ER (Fig 6A). We did detect minor alterations in the ASI axon-adjacent punctate pattern of wild-type DAF-28::mCherry in these animals (Fig 6A and 6B), which became less regular.

Next, we generated a DAF-28(R37C)::mCherry transgenic strain. We found that this transgene phenocopied the endogenous *sa191* mutation, as it caused severe developmental delay in the *daf-28(tm2308)* deletion background (Fig 6C). Imaging revealed dramatic differences in localization of DAF-28(R37C)::mCherry mutant protein as compared to wild-type. First, the protein appeared to strongly accumulate in the pharyngeal muscle and, to a lesser extent, in hypodermal tissue in the head, two tissues known to express *daf-28* (Fig 6D, 6E and 6F). DAF-28(R37C)::mCherry also appeared to accumulate in the ASI proximal axons, forming large aggregate-like puncta (Fig 6E–6H, block arrows). Unlike the puncta seen with the wild-type DAF-28::mCherry protein, these puncta were contained within the neuronal processes (Fig 6F and 6G). Strikingly, these puncta created voids in the fluorescence of soluble cytosolic GFP expressed in the ASI neurons, suggesting that the transgenic mutant protein disrupts axonal architecture of these cells (Fig 6H). Surprisingly, at least some of the DAF-28(R37C)::mCherry mutant protein was secreted, as evidenced by its presence in coelomocytes as early as L1 stage (Fig 6D, cc). Our data thus show that endogenous DAF-28(R37C) protein causes mild axonal defects, while overexpression of the DAF-28(R37C)::mCherry transgene, in addition to its own mistrafficking and accumulation, causes significant disruption of the ASI axons.

We took advantage of the mCherry-tagged DAF-28(R37C) to ask if the R37C mutation was indeed causing oxidative misfolding of this IGF-like protein. Misfolded MIDY insulins are known to engage in abnormal disulfide-linked protein complexes, resulting in loss of their detection as discrete species under non-reducing conditions [33]. DAF-28(R37C)::mCherry protein similarly did not resolve into any predominant bands under non-reducing conditions, while treating the worm lysates with reducing reagents produced a single band of expected size, approximately 37 kDa (Fig 6I, white arrow). As control, mCherry protein alone resolved into a single band under both reducing and non-reducing conditions (Fig 6I, black arrows). Although this is consistent with DAF-28(R37C)::mCherry protein being abnormally engaged in intermolecular disulfide-linked protein complexes, we could not unambiguously conclude this, since our ability to detect the wild-type DAF-28::mCherry protein, which, unlike the mutant, does not accumulate in any tissues, was not reliable (S3 Fig).

Finally, we examined the functionality and localization of the DAF-28::GFP protein, since we have found that it is not efficiently secreted (Fig 5C and 5D). DAF-28::GFP protein exhibited a strong toxic gain-of-function phenotype, causing severe developmental delay in the *daf-28(tm2308)* deletion background (Fig 6C). Moreover, DAF-28::GFP accumulated in both the cell body and axons of the ASI and ASJ neurons, eventually filling the cell bodies (Fig 6J–6L). This is consistent with misfolding and ER retention of a GFP-tagged secretory protein, since GFP is known to undergo oxidative misfolding in the ER of mammalian cells due to the presence of two buried cysteine residues [56,57].

### DAF-7/ TGF-β protein is misrouted and accumulates in the proximal axon of the ASI neuron in animals with folding mutation in *daf-28*

**Fig 7:**
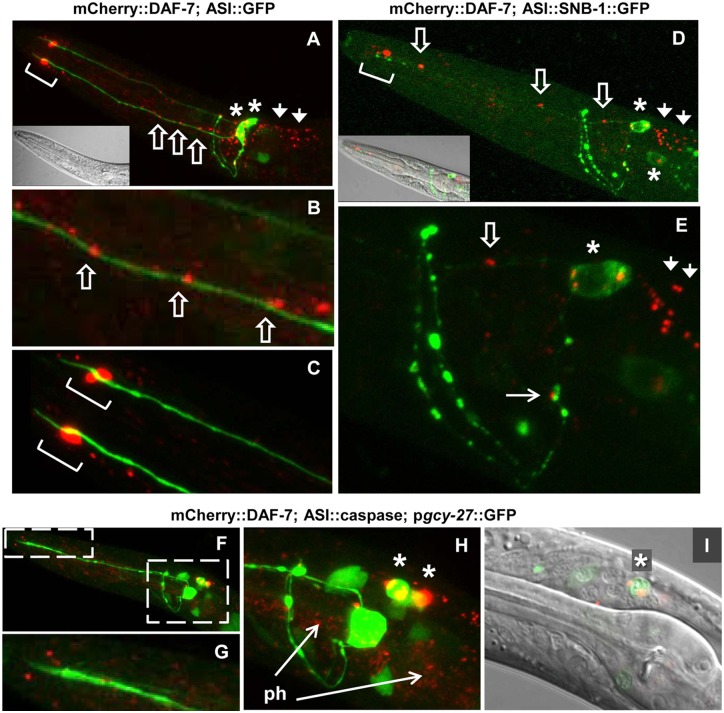
mCherry::DAF-7 protein is secreted from ASI cell body and trafficked to the distal end of the dendrite. **A-E**. mCherry fluorescence (red) can be seen in (stars) and posterior to (arrowheads) neuronal cell bodies, in puncta along the ASI dendrites (block arrows), and at the base of ASI cilia (brackets). Lower intensity red fluorescence in A and B represents pharyngeal expression. Green fluorescence in A-C is p*daf-7*::GFP,*rol-6* transgene (*ksIs2)*, used to visualize the ASI neuron. B and C are close-ups of A. Green fluorescence in D and E is synaptobrevin fusion protein (SNB::GFP) expressed in ASI, used to indicate synaptic densities in ASI axons. Only single small puncta of mCherry::DAF-7 can be seen in proximal region of ASI axon, with the majority of protein targeted to dendrite and posterior to cell body. E is a close-up of D. **F-I**. Green, p*gcy-27*::GFP transgene, expressed in ASI, ASJ and ASK neurons. Expression of activated caspase in the ASI neuron collapses the mCherry::DAF-7-derived fluorescence in the ASI cell body (star), and strongly decreases dendritic fluorescence, indicating their origin in the ASI neuron. The pharyngeal mCherry signal was not affected (ph, arrows). I is a single plane image.

Our data so far indicate that neither of the two most straightforward mechanisms—UPR induction or dominant-negative effect on wild-type insulin/IGF-like proteins—completely explain the gain-of-function phenotype caused by DAF-28(R37C) protein. As we observed defects in *daf-7* signaling in *sa191* animals (Fig 2), we next asked whether localization or secretion of our functional mCherry::DAF-7 protein was affected by the presence of the endogenous DAF-28(R37C) protein. In the wild-type background, mCherry::DAF-7 fluorescence was detected in neurons as well as in the pharyngeal muscles (Fig 7A–7E). In neurons, we observed punctate fluorescence in the cell bodies and in the area posterior to the ASI cell body (Fig 7A, 7D and 7E, stars and arrowheads, respectively). In contrast to DAF-28::mCherry, which was present in axons but excluded from dendrites, mCherry::DAF-7 protein was mainly detected along the dendrites of the ASI neurons (Fig 7A–7E, block arrows). Only rare small puncta were noted in the proximal axons (Fig 7E, arrow). Consistent with dendritic trafficking, we observed a large accumulation of the fluorescent signal surrounding the base of the ASI sensory cilia located at the distal end of the dendrite (Fig 7A, 7C and 7D, square brackets).

Although the maturation and trafficking of DAF-7/TGF-β have not been characterized in *C*. *elegans*, the signal outside the cilia could represent locally secreted mCherry::DAF-7 protein. To test this, we used a transgene that expresses active caspase specifically in the ASI neurons [58]. Activation of apoptosis in the ASI neurons collapsed both the ASI cell body- and posterior-localized fluorescent signal and strongly decreased the accumulation of mCherry fluorescence near cilia (Fig 7F–7I), confirming that they all represent the mCherry::DAF-7 protein secreted from the ASI neuron. ASI apoptosis did not eliminate the pharyngeal mCherry fluorescence (Fig 7H), suggesting that DAF-7 protein may also be expressed in the pharynx.

**Fig 8:**
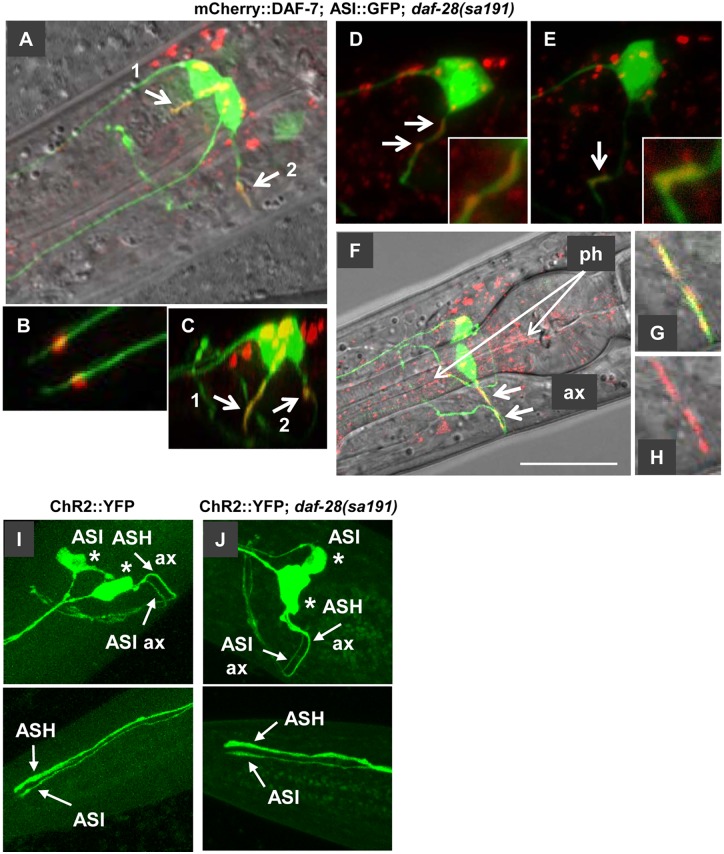
mCherry::DAF-7 protein accumulates in proximal axons of ASI neurons in animals carrying *daf-28(sa191)* mutation. **A-G**. Images of *sa191* animals expressing mCherry::DAF-7 protein, with p*daf-7*::GFP,*rol-6* transgene highlighting ASI neurons. A-C, L1 animal. D, E, L2, two sides of same animal. F-H, L4. Accumulation of mCherry::DAF-7 is indicated by bold arrows. Fluorescence in A-F are reconstructed z-stacks, G and H are single plane images, H is red channel only. C is a rotated image of A, showing red signal filling the proximal part of an ASI axon; B shows the mCherry::DAF-7 protein at the base of the cilia. mCherry signal also accumulates in the pharynx of L4 *sa191* animals (F). Scale bar in F is 20μm. **I,J**. ChannelRhodopsin2::YFP fusion protein normally localizes to the cell membranes of axons and dendrites of ASI neurons in *sa191* L4 animals. Upper panels, cell bodies (stars) and axons (ax) of ASI and ASH neurons. Lower panels, tips of the dendrites including cilia.

When placed in the background of the *daf-28(sa191)* mutation, mCherry::DAF-7 protein did not accumulate in the ASI cell bodies (Fig 8A–8F), and we did not detect any severe defects in the dendritic targeting (Fig 8B). Strikingly, unlike in a wild-type background, mCherry::DAF-7 protein accumulated in the proximal regions of the ASI axons in *sa191* mutant animals (Fig 8A–8H). This mistargeting of the mCherry::DAF-7 protein was evident already in the L1 (Fig 8A and 8C) and early L2 (Fig 8D and 8E) larval stages, which is the time the DAF-7 function in *sa191* animals is compromised (Fig 2A and 2B). This was not due to a generic trafficking defect, as localization of an unrelated secretory protein ChannelRhodopsin-2::YFP [59] to dendrites and axons was not affected by *sa191* (Fig 8I and 8J), and the shape of ASI cilia, which depends on the cellular trafficking, was also normal (Fig 8B and 8J, bottom panel). Thus, the mistargeting of DAF-7 to the axon of the ASI neuron is a specific molecular consequence of the expression of misfolded DAF-28(R37C) in the same cell, and may reflect the molecular events underlying the gain-of-function mechanism in *sa191* animals.

To quantify the mistargeting, we scored L1 or L2 animals with one or both of the mCherry-positive ASI neurons visualized in their entirety. In *daf-28(sa191)* mutant animals, 5 out of 9 ASI neurons examined had accumulated mCherry::DAF-7 protein in their axons, while 0 out of 8 ASI neurons in animals with wild-type *daf-28* had such axonal accumulation. This was not simply due to the *daf-28(sa191)* background being sensitizing to dauer entry, since we did not detect (0 out of 6) axonal mistargeting of mCherry::DAF-7 protein in the equally sensitized *daf-7(e1372)* animals. The accumulation of mCherry::DAF-7 in the ASI axon and in the pharynx was even more prominent in older *daf-28(sa191)* mutant animals (Fig 8F–8H, L4 animal shown). Based on these data, the folding mutation in the endogenous DAF-28/IGF protein leads to aberrant localization and accumulation of the wild-type bystander DAF-7/TGF-β protein in the axons of affected neurons.

**Fig 9:**
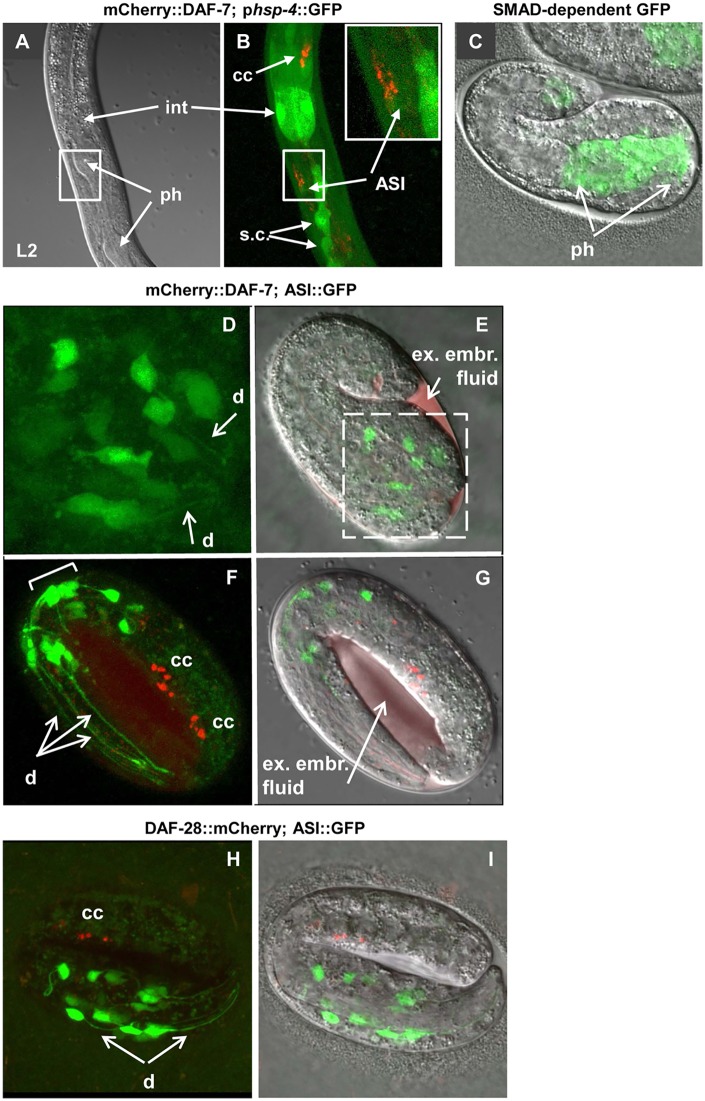
mCherry::DAF-7 is expressed in multiple neurons and secreted early in embryonic development. **A,B**. L2 animal expressing UPR reporter p*hsp-4*::GFP together with mCherry::DAF-7 protein. The UPR reporter is induced in the intestine and seam cells, but not in the ASI neuron. **C**. Comma-stage embryo expressing the SMAD::GFP reporter. The area of developing pharynx is indicated. **D-G**. Comma-stage (D,E) and 3-fold (F,G) embryos expressing mCherry::DAF-7 protein and p*daf-7*::GFP,*rol-6* transgene (ASI::GFP). GFP fluorescence can be seen in multiple cells in the head region of developing comma-stage embryo, and secreted mCherry::DAF-7 protein has already accumulated in the extraembryonic fluid at this stage. D is a close-up of the boxed area in E, green channel only. Bracket in F indicates position of amphid sensory neurons, including ASI. Note a strong mCherry fluorescence in coelomocytes in three-fold embryo (F,G). E and G are single plane images. **H,I**. 2.5-fold embryo expressing DAF-28::mCherry protein together with ASI::GFP transgene. DAF-28::mCherry protein is visible in coelomocytes. I is a single plane image.

If the ectopic mCherry::DAF-7 protein is mislocalized in the ASI neurons of *sa191* animals, how does it rescue the dauer phenotype? First, it is possible that overexpression of mCherry::DAF-7 protein results in the UPR induction and increased chaperone expression in the ASI neuron. We consider it unlikely, as we did not detect significant induction of the UPR reporter in the ASI neurons of L1-L2 animals expressing mCherry::DAF-7 (Fig 9A and 9B). It is also possible that the protein fraction that is still correctly localized to the ASI dendrite or secreted from its cell body is sufficient to produce the necessary pro-growth signaling. However, since we obtained much stronger rescue with the protein expressed from p*daf-7* promoter than with ASI-specific expression (Fig 2D), we asked whether DAF-7 protein may be expressed in cells other than the ASI neuron and other DAF-28-expressing cells, or at an earlier time than the mutant DAF-28(R37C) protein. Indeed, the SMAD::GFP reporter activity is clearly detectable in the developing pharynx already in the early embryonic stages (Fig 9C, comma-stage embryo). We detected expression of the established *daf-7* promoter-GFP transcriptional reporter *ksIs2* in multiple developing neurons in comma-stage embryos, as well as in several neurons in 3-fold embryos (Fig 9D–9G). Importantly, we detected accumulation of secreted mCherry::DAF-7 protein in the extraembryonic fluid at the same embryonic stages as the SMAD::GFP fluorescence (Fig 9E, comma stage shown), suggesting that DAF-7 activity may have a physiological role in the early embryos. We also detected pharyngeal expression and localization to coelomocytes in 3-fold embryos (Fig 9F and 9G). However, the earliest we were able to detect DAF-28::mCherry protein was in the coelomocytes of 2.5-fold stage embryos (Fig 9H and 9I). Thus, the rescue of *daf-28(sa191)* dauer phenotype by the overexpressed mCherry::DAF-7 protein could be due to its secretion from cells other than the ASI neurons, or due to its expression prior to the onset of DAF-28(R37C) expression.

### Two branches of UPR differentially affect the mislocalization of mCherry::DAF-7 protein in animals with folding mutation in *daf-28*

**Fig 10:**
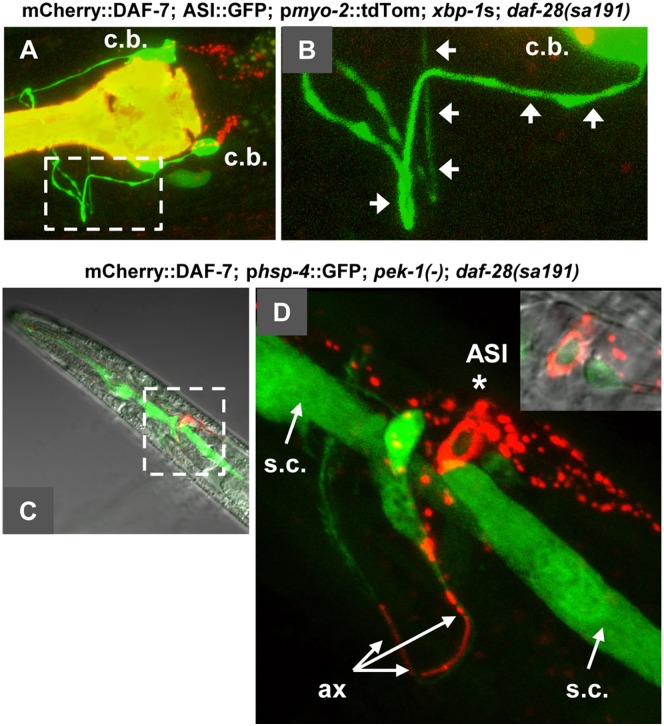
Expression of spliced XBP-1 and deletion of PERK have divergent effects on the localization of mCherry::DAF-7 protein in *daf-28(sa191)* animals. **A,B**. XBP-1s rescues the mislocalization of DAF-7. B is a close-up of the boxed area in A, arrowheads point to the proximal regions of ASI axons, devoid of red fluorescence. Strong mCherry::DAF-28 localization can be seen in puncta posterior to (and derived from) the ASI cell bodies in A. The bright yellow signal in A is the fluorescence of the tdTomato co-injection marker present in the *xbp-1*s array, that is expressed in the pharynx. **C,D**. Loss of PERK results in massive accumulation of mCherry::DAF-7 protein in *daf-28(sa191)* animals. Note the filled-in appearance of red fluorescence in the ASI cell body (star), prominent accumulation of the punctate signal posterior to the cell body, and continuous signal throughout the proximal axonal region and the beginning of the distal region of the ASI axon (ax). The green fluorescence in C,D is the UPR reporter used here to visualize neurons. Note also the UPR reporter induction in the nucleus of ASI, visible in the single-plane image in the inset in panel D, and in other neurons. s.c., seam cells.

Finally, we asked whether expression of spliced XBP-1 and deletion of *pek-1* rescued the *daf-28(sa191)* gain-of-function dauer phenotype by relieving the mislocalization of DAF-7 protein in the ASI neurons. Introduction of spliced XBP-1 into mCherry::DAF-7;*daf-28(sa191)* animals significantly reduced the mislocalization of mCherry::DAF-7 protein (Fig 10A and 10B). Of 14 axons examined, we found only two, in the same animal, with significant axonal localization, and additional two with intermediate phenotype. Together with the rescue of SMAD::GFP induction in *sa191* animals (Fig 4B), these data suggest that expression of spliced XBP-1 indeed rescues the bystander targeting of DAF-7 by the mutant DAF-28.

In contrast to the spliced XBP-1, introduction of *pek-1* deletion allele into mCherry::DAF-7;*daf-28(sa191)* animals not only did not rescue, but appeared to enhance the DAF-7 mislocalization (Fig 10C and 10D). The ASI proximal axons, and even some of their synaptic regions, appeared filled with red fluorescence (Fig 10D). Strikingly, mCherry::DAF-7 protein showed massive accumulation in the ASI neuronal cell bodies in these animals (Fig 10D, star). These data agree with the previous conclusion from Fig 4D that *pek-1(-)* may rescue the dauer phenotype of *daf-28(sa191)* animals by mechanism other than increasing DAF-7 activity. Therefore, although both UPR branches tested here are able to modulate the gain-of-function phenotype of the folding mutation in DAF-28, they appear to lead to vastly different molecular outcomes on the bystander target protein, DAF-7.

## Discussion

We have demonstrated that biogenesis of some proteins in the secretory pathway can be disrupted by misfolding of an unrelated endogenous protein, prior to the UPR induction or global disruption of ER proteostasis. In contrast to ectopically (over)expressed misfolded disease proteins, where re-distribution of general chaperones leads to the global disruption of essential cellular functions, we observe a different bystander mechanism, where a specific and targeted effect on either folding, maturation or trafficking of a specific non-mutant protein precedes the global ER stress and explains the selectivity of the resulting phenotype.

### Misfolding of DAF-28(R37C) protein as a likely proximal cause of its gain-of-function phenotype

Our data support oxidative misfolding of the mutant DAF-28 protein as the most proximal cause. As expected for a folding mutation [60], altering the overall folding capacity of the ER modulated the penetrance of the dauer phenotype caused by the endogenous DAF-28(R37C) mutation, while the transgenic mutant protein accumulated in tissues and exhibited aggregation-like behavior. Interestingly, as judged by its uptake into coelomocytes, at least some of the DAF-28(R37C)::mCherry mutant protein was secreted. ER quality control mechanisms typically prevent secretion of misfolded or non-native proteins. However, classical amyloid diseases are associated with secretion of destabilized amyloidogenic proteins, and increasing the stringency of the ER quality control by activation of the UPR transcription factor ATF6 selectively decreases their secretion [61]. The mechanism by which some of the DAF-28(R37C)::mCherry protein evades ER quality control is unclear. One possibility is that incorrect disulfide bond pairing in a small insulin/IGF-like mutant protein may result in an alternative structure that, while non-native, may present as a globular compact protein. Indeed, mammalian IGF is known to form two alternative stable conformations *in vitro*, only one of which has the correct disulfide bond arrangement and is active [62], and a non-native mammalian mini-proinsulin has been shown to bypass ER quality control and be secreted in yeast [63].

Misfolded insulin, encoded by PNDM/MIDY mutations, can affect secretion of the wild-type insulin from the same cell [32,33]. On the other hand, Rajan *et*. *al*. [55] found that some insulin mutants, including PNDM-associated R89C, do not interfere with secretion of the wild-type insulin. Both R89C human insulin mutation and the *C*. *elegans* R37C mutation in DAF-28 introduce an unpaired cysteine while also disrupting the proteolytic processing site. Interestingly, similar to our DAF-28(R37C)::mCherry, the R89C mutant insulin was partially targeted to secretory granules and secreted, even though it strongly activated ER stress response and expression of the pro-apoptotic protein CHOP [55]. We did not detect ER retention of the wild-type DAF-28::mCherry protein in animals with *sa191* mutation and observed only mild alterations in its axonal localization. A detailed biochemical characterization will be necessary to fully understand the molecular consequences that expression of DAF-28(R37C) has on either wild-type DAF-28 or other insulin/IGF-like proteins.

### UPR signaling pathways can modulate the gain-of-function developmental phenotype caused by DAF-28(R37C) protein

Our second observation is that, while activation of UPR plays a complex role in the phenotypic outcome of the *sa191* mutation, it is unlikely to be its triggering mechanism. At the time *sa191* mutation exerts its phenotypic effect (L1/early L2 stages), the UPR reporter activity in the ASI neurons was either not induced, or was similar to that in several other neurons and much weaker than its induction in cells of animals carrying *pek-1* deletion. Overexpression of the spliced XBP-1 showed that forced activation of this adaptive UPR branch can attenuate dauer signaling in *sa191* animals, by suppressing the bystander effects of the mutant DAF-28 on localization and/or activity of DAF-7. Deletion of *pek-1*, on the other hand, prevented commitment to dauer by *sa191* animals under non-stressful growth conditions, as was reported at high temperature [18]. Thus, unlike activation of IRE1/XBP1 branch of UPR, activation of PERK actually enhances the dauer phenotype of *sa191* animals. However, about 20% of *pek-1*-deficient *sa191* animals still abnormally entered the pre-dauer L2d stage at higher population densities (Fig 4D), indicating that translational silencing or any transcriptional outcomes of the PERK activation did not, by themselves, initiate the molecular events leading to *sa191* gain-of-function phenotype. These observations are inconsistent with the UPR being the trigger for ASI-specific dysfunction. The UPR appears to modulate the strength of signaling of TGF-β and insulin/IGF-like pathways, by affecting the balance of the folding environment in the ER and possibly impacting on translation. We suggest that, in context of the endogenous *sa191* mutation, activation of the PERK/eIF2α pathway could amplify the initial trigger, which we infer to be the interference with the normal biogenesis of DAF-7/TGF-β by the folding mutant of DAF-28/IGF. However, PERK could also exert its effects through a yet-unknown, ASI- or DAF-7-independent mechanism, since we found that, despite its ability to decrease dauer phenotype, deletion of *pek-1* enhanced the mislocalization of the transgenic mCherry::DAF-7 protein in animals with DAF-28(R37C) mutation, and caused its massive intracellular accumulation in the ASI neurons. This was reminiscent of the findings that the PERK-deficient pancreatic β-cells exhibit severe disruption of ER-Golgi anterograde trafficking and disruption of the Golgi complex, while, paradoxically, decrease of PERK gene dosage ameliorated the progression of the *Akita* mouse expressing the gain-of-function insulin mutant to overt diabetes; the latter was proposed to be due to decrease degradation of the wild type insulin [64]. Our findings highlights the complexity of the UPR effects and underscore the need to further understand the role of UPR signaling under chronic, physiological, stress conditions.

### A model for specific bystander targeting of endogenous DAF-7/TGF-β

**Fig 11:**
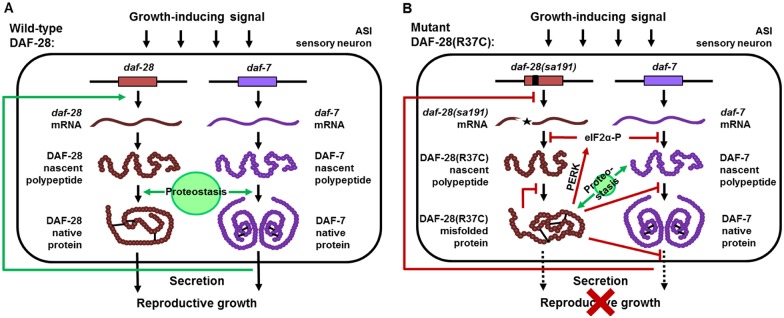
A model for bystander targeting of DAF-7/TGF-β. **A**. In wild-type animals, growth-inducing environment is sensed by the ASI neuron, resulting in secretion of pro-growth proteins DAF-28 and DAF-7. DAF-7 regulation of *daf-28* transcription creates a feed-forward loop (green arrow). Both DAF-28 and DAF-7 may require proteostasis components for their biogenesis. **B**. DAF-28(R37C) mutant protein interferes with folding, maturation, or secretion of endogenous DAF-7 (red inhibitory arrows), likely by titrating the necessary proteostasis component(s). It may also affect other pro-growth insulin/IGF proteins. The resulting combined decrease in secreted functional DAF-28 and DAF-7 proteins, together with the feed-back transcriptional regulation of *daf-28*, shift the growth *vs*. dauer signaling balance towards dauer entry. The presence of the misfolded species also activates PERK (red arrow), leading to translational attenuation of DAF-28 (or other pro-growth insulin/IGF proteins) and DAF-7, amplifying the signal and ensuring the commitment to dauer entry. Overexpression of XBP-1s improves pro-growth signaling by increasing availability of ER chaperones and other proteostasis machinery (green arrows), thereby reducing misfolding of DAF-28 and DAF-7 and/or their competition for the necessary proteostasis component(s).

The selective targeting of DAF-7/TGF-β protein biogenesis, in particular its mislocalization to the proximal axon, is an intriguing finding. As previously noted, *sa191* animals phenotypically resemble animals with deficient *daf-7* signaling [17], and we found that decreased DAF-7 function explains the *sa191* gain-of-function dauer phenotype. However, because transcription of *daf-7* does not depend on insulin signaling, the mechanism by which DAF-7 activity might be affected in these animals was unclear. Our data suggest the following model for the bystander targeting of DAF-7 protein and for its phenotypic outcome in *sa191* animals (Fig 11).

First, misfolded DAF-28(R37C) protein in *sa191* animals may titrate a component of the ER/Golgi proteostasis machinery that is required for efficient folding or maturation of the endogenous DAF-7 protein. This could be a chaperone required for productive folding by both DAF-7 and wild-type DAF-28. Alternatively, DAF-7 may only share such a chaperone with the misfolded and not with wild-type DAF-28. The latter is more likely, as we did not detect a strong effect of *sa191* mutation on the wild-type DAF-28. Because of the role of the unpaired cysteine, and the knotted arrangement of the disulfide bonds in DAF-7, a potential candidate for this competition may be an oxidoreductase [65,66]. Another example of a potential candidate could be a folding chaperone with restricted client repertoire. For example, mammalian IGF proteins are completely dependent on the selective ER chaperone GRP94 for folding and secretion [16,19,20].

The resulting defects in the folding or maturation of the DAF-7 protein lead to its striking mislocalization and accumulation in the proximal axon of the ASI neuron. Axonal trafficking defects are common in neurodegeneration, yet, how soluble cargo is selectively sorted to the vesicles destined to axons *vs*. dendrites is not understood. It is possible that non-native species of DAF-7, generated in the presence of misfolded DAF-28(R37C), interact abnormally with the sorting machinery, resulting in mistrafficking.

The combined outcome of these defects in DAF-7 folding, processing, and/or trafficking, together with decreased function of the mutant DAF-28(R37C) protein, result in decreased pro-growth signaling, despite the normal perception of the food signal (Fig 11B). Decreased *daf-7* activity, in turn, affects transcription of pro-growth insulin/IGF genes [17,67], further reducing the pro-growth signaling. This balance of growth *vs*. dauer signals may result in activation of the dauer program, but may not be sufficient for the full commitment to dauer, as many *sa191* animals that enter the L2d stage quickly resume reproductive growth. We suggest that PERK signaling is necessary for the dauer commitment of *sa191* animals because it provides amplification of the signal, perhaps through translational attenuation (Fig 11B).

If the defect in DAF-7 protein biogenesis in *sa191* animals is mediated by titration of a necessary chaperone by misfolded DAF-28(R37C), how can overexpression of DAF-7 rescue this defect? Overexpression of a protein under conditions of chaperone insufficiency would further increase the folding stress in the ER; yet, we observe a strong rescue of the *sa191* dauer phenotype by overexpressed DAF-7 (Fig 2D). Our data suggest several explanations. First, *daf-7* is thought to be expressed predominantly in the ASI and, in certain conditions, the ASJ and a subset of mechanosensory neurons [68]. However, we find that in embryos and just-hatched L1 larvae, *daf-7* is expressed in multiple neurons. If these neurons do not express *daf-28(sa191)*, they may be a source of native, functional secreted DAF-7 transgenic protein. Second, we observe expression and secretion of mCherry::DAF-7 protein already in the early stage embryos and throughout subsequent embryonic development, while DAF-28::mCherry is expressed later. Similarly, the ASI-specific *gpa-4* promoter in ASI::DAF-7 transgene is known to be active in early embryos [69]. Thus, transgenic mCherry::DAF-7 may be secreted prior to the disruptive effects of the endogenous DAF-28(R37C) on its biogenesis. This interpretation is supported by the maternal rescue of dauer signaling by the transgenic DAF-7, which suggests that the presence of this protein only in the embryo, without further expression in larval stages, is sufficient to prevent dauer induction.

### Targeted rather than global bystander mechanism explains the specific phenotype of a broadly expressed misfolded protein

What accounts for the specificity of the toxic effect of the misfolded DAF-28(R37C) protein, which appears to only target a dauer-promoting function in the ASI neuron despite being expressed in many other cells? The most trivial possibility is that the mutant DAF-28 protein may be expressed at higher levels in the ASI neuron than in other cells in the early larval stages, although this is not supported by transcriptional reporters. Second, the proteostasis component(s) required for both DAF-28(R37C) and DAF-7 protein biogenesis may only be limiting in the ASI neuron but present in a sufficient amount to buffer the misfolded DAF-28 mutant in other cells. In this case, the observed lack of generic protein mislocalization and of dysfunction or degeneration of the ASI neuron would also imply that the requirement for this chaperone is not as strong for other proteins expressed in this cell as it is for DAF-7.

An intriguing possibility is that the ASI neuron could, due to a yet unknown intrinsic property, be selectively sensitive to misfolding in the ER. Mammalian Purkinje cells, for example, are selectively sensitive to mutations in several broadly or ubiquitously expressed proteins, which cause ER stress and toxicity in Purkinje cells but not in other neuronal cell types [70–72]. Alternatively, sensitivity of dauer activation to ER stress, rather than selective sensitivity of the ASI neuron, could be responsible for the ASI-specific dauer phenotype, for example due to being tuned to small changes in the rate of secretion of DAF-28 and DAF-7. Indeed, DAF-28 and DAF-7 cooperate to activate a growth-promoting feed-forward loop, while a decrease in DAF-7 feeds back to attenuate *daf-28* transcription (Fig 11).

Several observations, including some that were previously unexplained, support these two possibilities. First, a highly-expressed p*daf-7*::GFP transcriptional reporter *saIs8* was previously observed to abnormally induce dauer entry for unknown reasons [27]. Examination of its sequence suggests that it may be prone to misfolding since, in addition to the DAF-7 promoter, this reporter contains an N-terminal fragment of the DAF-7 protein, including ER signal sequence and most of its LAP, fused to a GFP moiety. In mammalian TGF-β, the LAP forms extensive hydrophobic contacts with the C-terminal cysteine-knot hormone domain [73], which is deleted in this reporter. Misfolding of this reporter in the ER of the ASI neuron may explain the observed dauer induction. Second, we find that a DAF-28::GFP fusion protein induces a strong gain-of-function dauer phenotype in *daf-28* deficient background, likely due to the oxidative misfolding of the GFP and the accumulation of the misfolded fusion protein in the ER of the ASI (and ASJ) neurons. Finally, it was previously reported that crossing DAF-28::GFP transgene into a *daf-8(m85)* mutant background unexpectedly resulted in irrecoverable dauer-constitutive phenotype [74]. DAF-8 is a pro-growth R-Smad transcription factor in the DAF-7/TGF-β signaling pathway. The sensitivity of the ASI neuron, and/or of the dauer signaling pathway, to protein misfolding in the ER may explain the dauer phenotype in all three of these examples.

Our data suggest that DAF-7/TGF-β protein is a sensitive and selective target of the bystander effect caused by misfolded DAF-28/IGF. It will be very important to understand what makes a particular protein a target for bystander misfolding [75], and whether different proteins are targeted by distinct misfolded species. Although global disruption of proteostasis and induction of stress responses are common to the toxic effects of misfolded proteins, there are examples of specific bystander targeting. In *Drosophila*, expression of human insulin bearing the Akita C96Y mutation results in ER stress and cellular dysfunction. While this mutant protein caused a general degeneration phenotype in the eye, its expression in the wing imaginal discs resulted in phenocopies of *Notch* and *crossveinless* mutations [76,77]. Interestingly, a targeted deletion of the ER thiol oxidase Ero1L specifically in the wing also caused a phenocopy of *Notch*, by inducing misfolding of Notch protein while not affecting other secretory proteins [78].

Our data suggest that a global *vs*. targeted bystander mechanism of a given folding mutation may depend on (1) the nature of the misfolded species it produces, (2) the identity, client repertoire, and availability of chaperones that bind these misfolded species, and, importantly, (3) the presence in the same cell of susceptible bystander targets controlling sensitive cellular or organismal processes. Identifying these potential bystander targets and the restricted chaperones could be instrumental in understanding—and protecting against—the specific toxic phenotypes in protein misfolding diseases.

## Materials and Methods

### Strains and genetics

Standard methods were used for worm culture and genetics [79]. Animals were synchronized by picking gastrula-stage embryos, or by hypochlorite treatment for FACS analysis.

The following strains were obtained from the *Caenorhabditis* Genetics Center (CGC): JT191(*daf-28(sa191)*V), TY3862(*daf-7(e1372)*III;*cuIs5*[p*myo-2*C::GFP + pRF4(*rol-6(su1006)*)]), VC1099(*hsp-4(gk514)*II), RB545(*pek-1(ok275)*X), SJ4005(*zcIs4*[p*hsp-4*::GFP]V), PD4792(*mIs11*[p*myo-2*::GFP + p*pes-10*::GFP + gut-promoter::GFP]IV), HT2099(*unc-119(ed3)* III;*wwEx85*[p*daf-28*::GFP]), FK181(*ksIs2*[p*daf-7*::GFP + *rol-6(su1006)*]), NM440(*unc-104(e1265)*II;*jsIs1*[pSB120(*snb-1*::GFP);pRF4 (*rol-6(su1006)*)]).

N2AM (wild-type) is a subclone of N2Bristol from Morimoto Lab. DAF-28::GFP strain (*svIs69* array) was from Naredi Lab (U. of Gothenburg). Strain 2308(*daf-28(tm2308)*V) was from the National BioResource Project (Japan). ZM7963(*hpDf761*II;*daf-28(tm2308)*V) and XL153(*ntIs27*[*p*sra-*6*::ChR2::YFP, *p*unc-*122*::dsRed]) were a gift from Fang-Yen Lab (UPenn).

Crosses were conducted using phenotypic or fluorescent chromosomal markers (http://www.wormbuilder.org/). Strains were confirmed by PCR and restriction digest or sequencing.

### Transgenes construction

Transgenes were injected by Knudra Transgenics (USA) as 20ng/μL plasmid DNA and 80ng/μL sonicated salmon sperm DNA.

All PCR products were verified by sequencing. Restriction sites were introduced into PCR primers.

### *drxEx20* [p*daf-7*::mCherry::*daf-7*::*unc-54* 3’UTR]

A 2.6 kb NaeI/XbaI fragment containing the *daf-7* promoter region and first 23 residues of the DAF-7 protein, and a 1278bp SacI/EagI fragment from amino acid 23 to stop codon were amplified from N2 genomic DNA. XbaI/SacI worm mCherry minus the stop codon was amplified from pCFJ104 (Addgene #19328). The three fragments were assembled to exchange the p*myo-3*::mCherry in pCFJ104.

### *drxEx21* [p*daf-28*::*daf-28*::mCherry::*unc-54* 3’UTR]

The 703bp PstI/XbaI fragment containing coding region of *daf-28*, and the 2.0 kb SphI/PstI *daf-28* promoter region [21] were amplified from N2 genomic DNA. The 934bp XbaI/SacI worm mCherry and the 873bp SacI/PvuII *unc-54* 3’UTR fragments were amplified from pCFJ104. Fragments were assembled in pMCS5 plasmid.

### *drxEx22* [p*daf-28*::*daf-28(sa191)*::mCherry::*unc-54* 3’UTR]

The coding region of *daf-28* was amplified from *daf-28(sa191)* genomic DNA as PstI/XbaI fragment and exchanged with the wild-type coding region of *drxEx21*.

### Developmental assays

Animals were grown on fresh plates seeded with OP50 *E*. *coli* at 20°C under non-crowded/non-contaminated conditions for at least 2 generations prior to embryo picking, to avoid effects on dauer entry [80]. 20–40 YA animals were allowed to lay embryos for 24 hours at 20°C. For transgenic rescue assays, only transgenic (parent) animals were picked, so that the non-transgenic animals among their progeny were siblings to the transgenic ones. From these, 100–200 gastrula-stage embryos were picked onto new plates and allowed to develop for 65–66 hours at 20°C. Animals with embryos present *in uteri* were scored as reproductive adults; YA or late L4 stages (based on gonad development) were scored as mildly delayed; and early L4 or earlier stages (mainly L2d and/or dauers) as severely delayed. Dauer larvae were radially constricted, lacked pharyngeal pumping, and had visibly constricted pharynxes [80]. L2d larvae were radially constricted to a lesser extent than dauers, had a uniformly dark intestine (Fig 4F and 4H) [80] and exhibited slow pharyngeal pumping. All developmental assays were repeated at least three times, raw data are in the Supplemental Data Table.

For SMAD-reporter/development correlation, larvae were separated at ~35 hours post-gastrula into ‘bright’ and ‘dim’ populations based on GFP fluorescence viewed through stereo microscope, and allowed to develop for an additional 30 hours. Because of the separation, animals experienced drop in population density resulting in slight increase in reproductive development in *sa191*.

### FACS sorting

GFP intensity was measured in L1/early L2 animals using BioSorter (Union Biometrica, USA) for three strains carrying the *cuIs5* transgene, and non-transgenic N2AM. Data analysis was performed by FlowJo software. An initial gate was set as the measurement of length (time of flight) versus absorbance (extinction) to distinguish larvae from debris. All animals with GFP intensity values higher than maximum detected in the non-transgenic N2AM strain were included in analysis.

### Western blot

50 or 100 L2 larvae in 30μL of water were flash-frozen in liquid nitrogen. 15μL of reducing or non-reducing sample buffer was added and samples incubated at 85–95°C for 10 minutes. Protein amounts were verified by Ponceau stain of membranes. Anti-RFP (5F8, ChromoTek, Germany) was used to detect DAF-28(R37C)::mCherry. JT191 and TU3401 strains were used as negative and positive controls. For the blot in S3 Fig, full plates were collected and frozen in aliquots. Worms were lysed by mechanical disruption, as described in [8], treated with reducing/non-reducing sample buffer, and further processed as above.

### Swimming

10 L4 larvae (total 150 animals/strain) were picked into 25μL of M9 solution on a glass slide and acclimated for 1 minute. One minute movies were taken and analyzed using wrMTrck ImageJ plug-in (Dr. Jesper Pedersen, http://www.phage.dk/plugins/wrmtrck.html).

### Brood size

Pharyngeal GFP marker was introduced into *daf-28(sa191)* animals from PD4792 strain by crossing. PD4792 was used as control. 5 L4 larvae per plate (total 30 animals/strain) were plated and allowed to lay progeny at 20°C. Animals were transferred to new plates every 24-hours until egg-laying ceased. Number of progeny was quantified by counting fluorescent pharynxes using ImageJ.

### Body length

50 L4 larvae of each strain were plated on seeded plates and incubated for 24 hours at 20°C. 30 adult animals were immobilized on glass slides using 20mM sodium azide, and imaged at 50X magnification on Leica M205FA. Microscale was included in the images as a ruler. Body length was measured from the tip of the nose to the tip of the tail with ImageJ.

### Imaging

Confocal: animals were mounted on 2% agar pads with azide and imaged with Zeiss LSM700 microscope at Cell Imaging Center, Drexel University, using 1.4NA 63x oil objective. 12 bit confocal stacks were reconstructed in ImageJ as 3D projections, and where indicated overlaid on single plane DIC images.

Stereo: animals were mounted as above, or immobilized by chilling on plates. Imaging was performed with Leica M205FA microscope and Hamamatsu Orca R2 camera, keeping magnification and intensity of fluorescent sources (Chroma PhotoFluor 2) constant within experiment.

### Statistics

All Chi-square, ANOVA, and *t*-test analyses were performed using Prism software (GraphPad, USA). ANOVA was followed by multiple comparisons post-test, as indicated in Figure legend. α and significance levels are also indicated. For developmental assays, at least three replicates were used, the raw data including the number of animals per replicate are in Supplemental Data Table.

## Supporting Information

S1 Fig *daf-7* expression is unaffected in *sa191* animals. **A**. p*daf-7*::GFP expression in the ASI neuron is not attenuated in *daf-28*(*sa191*) mutant animals. Animals were imaged at L1/L2 larval stage. Fluorescence was quantified from two or three consecutive single plane confocal images taken through the middle of the neuronal cell body. ImageJ was used to quantify average fluorescence intensity. Error bars are mean±SD, each symbol corresponds to a single ASI neuron. **B**. Schematic representation of the mCherry::DAF-7 transgene, not to scale. *daf-7* sequence was cloned from N2AM genomic DNA. mCherry is fused to the N-terminus of the latency-associated peptide (LAP), which in TGF-β proteins binds extracellular matrix upon secretion, while still complexed with the mature growth factor cysteine knot domain, and becomes separated from it following activation. Thus, mCherry reports on the intracellular trafficking of the intact DAF-7 protein and the extracellular localization of DAF-7's LAP, whether still complexed with the growth factor domain or after its liberation. The location of the mature growth factor/cysteine knot domain is indicated. Data in A were analyzed by unpaired *t*-test with Welch’s correction, α = 0.05, ns = not significant.
(TIF)

S2 Fig PERK deletion does not prevent entry of *sa191* animals into L2d/pre-dauer stage at 20°C in larger populations. 20 young adults were picked per 6cm plate, and plates were examined three days later. All animals carry the p*hsp-4*::GFP reporter in the indicated backgrounds, the reporter is heterozygous in *pek-1* animals. Examples of L2d animals are indicated by red arrows.
(TIF)

S3 Fig Reducing/non-reducing PAGE analysis of DAF-28 proteins. **A**. Reducing conditions. **B**. Non-reducing conditions. DAF-28(R37C)::mCherry protein (white arrow, lane 4) is not resolved under non-reducing conditions, while a control mCherry protein (lane 2) is equally well-resolved under reducing and non-reducing conditions. The wild-type DAF-28::mCherry protein (red arrow, line 3) is expressed at too low levels to be reliably detected. Line 1, non-transgenic animals.
(TIF)

S1 Data Table (DOCX)
