# Supplementary figures and images for: A Bystander Mechanism Explains the Specific Phenotype of a Broadly Expressed Misfolded Protein
Source: PLoS Genet. 2016 Dec 7;12(12):e1006450. doi: 10.1371/journal.pgen.1006450 (PMC5142776; doi:10.1371/journal.pgen.1006450)

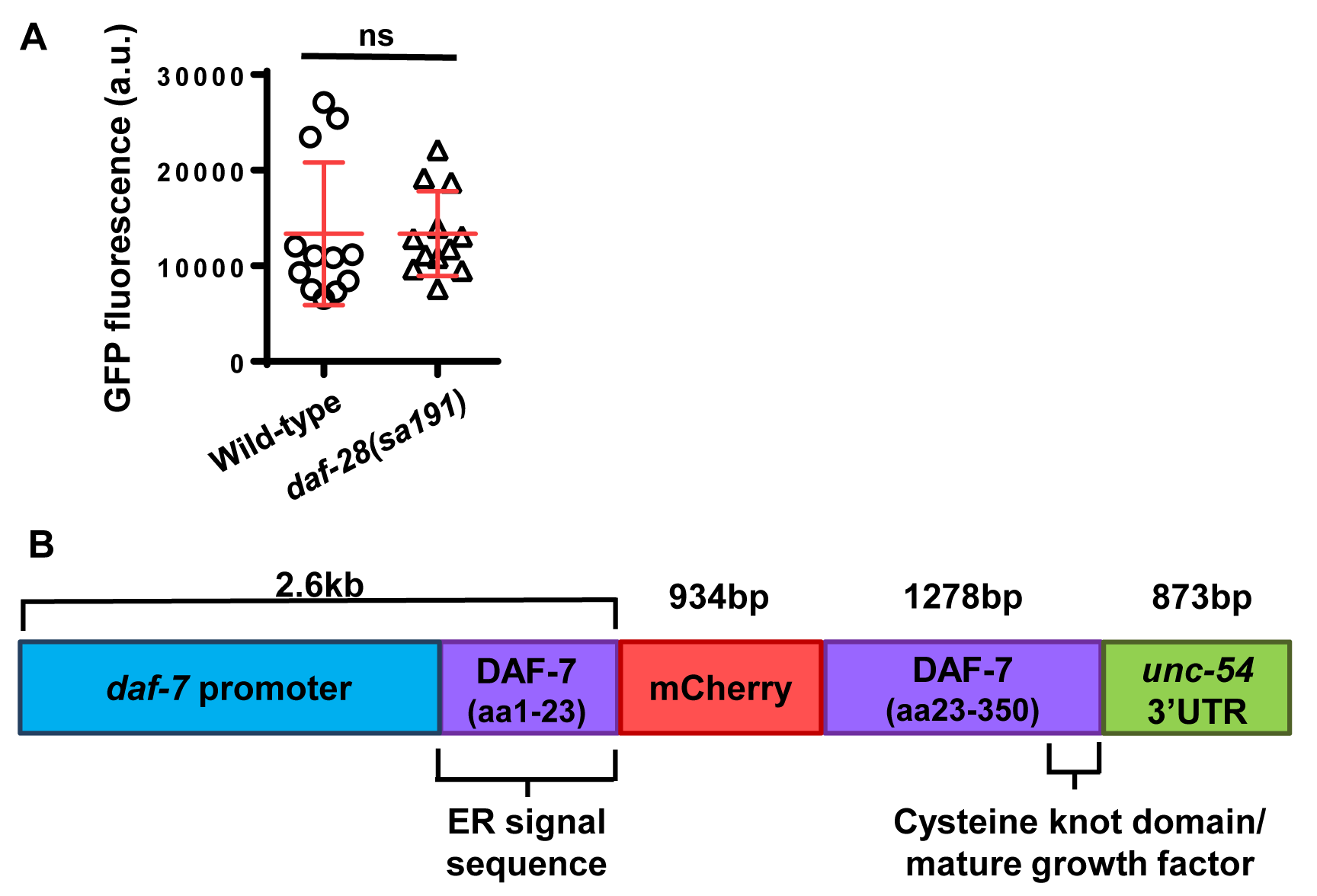

Supplement: S1 Fig — A. pdaf-7::GFP expression in the ASI neuron is not attenuated in daf-28(sa191) mutant animals. Animals were imaged at L1/L2 larval stage. Fluorescence was quantified from two or three consecutive single plane confocal images taken through the middle of the neuronal cell body. ImageJ was used to quantify average fluorescence intensity. Error bars are mean±SD, each symbol corresponds to a single ASI neuron. B. Schematic representation of the mCherry::DAF-7 transgene, not to scale. daf-7 sequence was cloned from N2AM genomic DNA. mCherry is fused to the N-terminus of the latency-associated peptide (LAP), which in TGF-β proteins binds extracellular matrix upon secretion, while still complexed with the mature growth factor cysteine knot domain, and becomes separated from it following activation. Thus, mCherry reports on the intracellular trafficking of the intact DAF-7 protein and the extracellular localization of DAF-7's LAP, whether still complexed with the growth factor domain or after its liberation. The location of the mature growth factor/cysteine knot domain is indicated. Data in A were analyzed by unpaired t-test with Welch’s correction, α = 0.05, ns = not significant. (TIF) [file pgen.1006450.s001.tif]

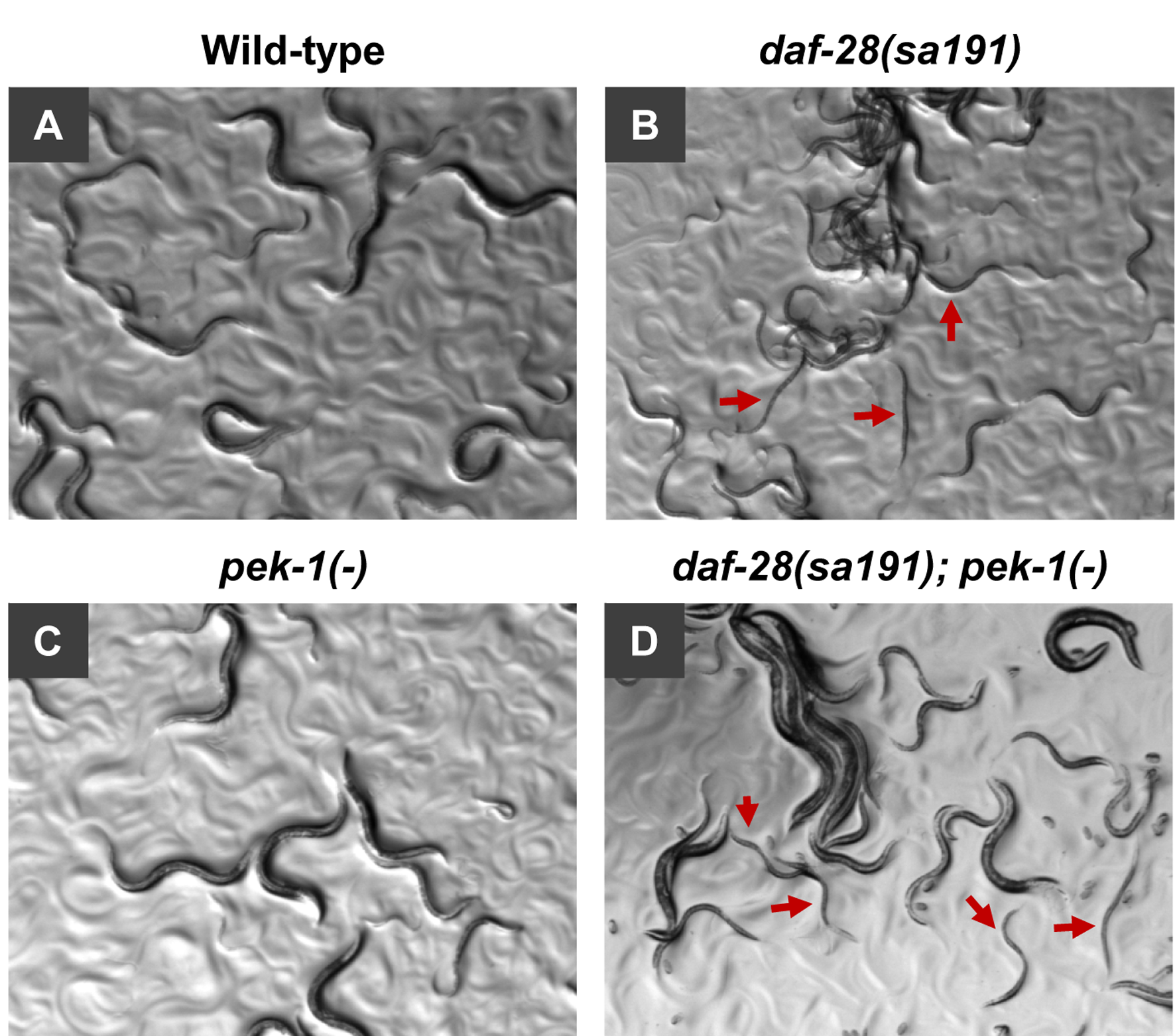

Supplement: S2 Fig — 20 young adults were picked per 6cm plate, and plates were examined three days later. All animals carry the phsp-4::GFP reporter in the indicated backgrounds, the reporter is heterozygous in pek-1 animals. Examples of L2d animals are indicated by red arrows. (TIF) [file pgen.1006450.s002.tif]

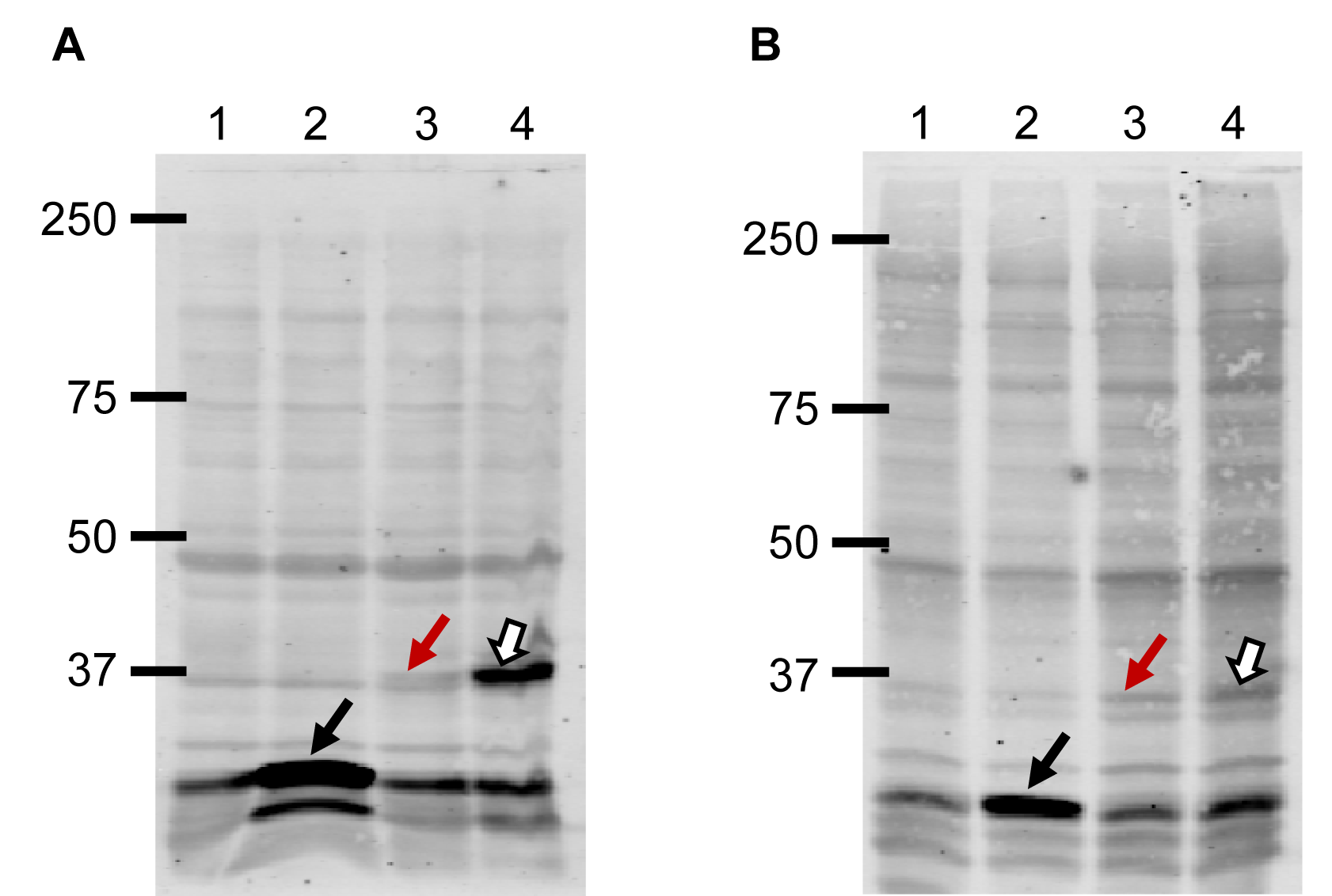

Supplement: S3 Fig — A. Reducing conditions. B. Non-reducing conditions. DAF-28(R37C)::mCherry protein (white arrow, lane 4) is not resolved under non-reducing conditions, while a control mCherry protein (lane 2) is equally well-resolved under reducing and non-reducing conditions. The wild-type DAF-28::mCherry protein (red arrow, line 3) is expressed at too low levels to be reliably detected. Line 1, non-transgenic animals. (TIF) [file pgen.1006450.s003.tif]
