# Supplementary material for: A Bystander Mechanism Explains the Specific Phenotype of a Broadly Expressed Misfolded Protein
Source: PLoS Genet. 2016 Dec 7;12(12):e1006450. doi: 10.1371/journal.pgen.1006450 (PMC5142776; doi:10.1371/journal.pgen.1006450)
Supplement: S1 Data Table — (DOCX) [file pgen.1006450.s004.docx]

**S4. Data Table.**

| **Strain** | **Genotype** | **Exp.** | ***n*** | **Reprod. adult** | **Mild delay** | **Severe delay** |
| --- | --- | --- | --- | --- | --- | --- |
| N2AM | Wild-type | 1 | 165 | 165 | 0 | 0 |
|  |  | 2 | 149 | 146 | 3 | 0 |
|  |  | 3 | 159 | 156 | 3 | 0 |
| JT191 | *daf-28(sa191)*V. | 1 | 141 | 3 | 70 | 68 |
|  |  | 2 | 144 | 3 | 108 | 33 |
|  |  | 3 | 131 | 1 | 109 | 21 |
| 2308 | *daf-28(tm2308)*V. | 1 | 144 | 139 | 5 | 0 |
|  |  | 2 | 105 | 90 | 8 | 7 |
|  |  | 3 | 95 | 86 | 7 | 2 |
| DAF-28::GFP | DAF-28::GFP | 1 | 117 | 20 | 95 | 2 |
|  |  | 2 | 141 | 3 | 135 | 3 |
|  |  | 3 | 104 | 25 | 74 | 5 |
| TGF102 | DAF-28::GFP;*daf-28(tm2308)*V. | 1 | 149 | 0 | 4 | 145 |
|  |  | 2 | 85 | 0 | 1 | 84 |
|  |  | 3 | 134 | 0 | 1 | 133 |
| TGF145 | *drxEx21*[p*daf-28*::*daf-28*::mCherry] (high) | 1 | 105 | 88 | 16 | 1 |
|  |  | 2 | 110 | 94 | 16 | 0 |
|  |  | 3 | 101 | 86 | 12 | 3 |
| TGF145 | Non-Tg sibling | 1 | 23 | 23 | 0 | 0 |
|  |  | 2 | 50 | 50 | 0 | 0 |
|  |  | 3 | 41 | 40 | 1 | 0 |
| TGF149 | *drxEx21*(high);*daf-28(tm2308)*V. | 1 | 95 | 87 | 8 | 0 |
|  |  | 2 | 100 | 88 | 12 | 0 |
| TGF149 | Non-Tg sibling | 1 | 16 | 16 | 0 | 0 |
|  |  | 2 | 49 | 47 | 1 | 1 |
| TGF097 | *drxEx21*(low);*daf-28(tm2308)*V. | 1 | 154 | 67 | 77 | 10 |
|  |  | 2 | 47 | 16 | 31 | 0 |
|  |  | 3 | 87 | 76 | 11 | 0 |
| TGF109 | *drxEx22*[p*daf-28*::*daf-28(sa191)*::mCherry] | 1 | 120 | 75 | 36 | 9 |
|  |  | 2 | 77 | 52 | 23 | 2 |
|  |  | 3 | 71 | 58 | 11 | 2 |
|  |  | 4 | 95 | 78 | 14 | 3 |
| TGF109 | Non-Tg sibling | 1 | 44 | 36 | 8 | 0 |
|  |  | 2 | 57 | 55 | 2 | 0 |
|  |  | 3 | 56 | 55 | 1 | 0 |
|  |  | 4 | 45 | 42 | 3 | 0 |
| TGF081 | *drxEx22*;*daf-28(tm2308)*V. | 1 | 94 | 38 | 27 | 29 |
|  |  | 2 | 60 | 22 | 26 | 12 |
|  |  | 3 | 45 | 15 | 15 | 15 |
| TGF081 | Non-Tg sibling | 1 | 64 | 45 | 19 | 0 |
|  |  | 2 | 49 | 37 | 12 | 0 |
|  |  | 3 | 89 | 65 | 24 | 0 |

| **Strain** | **Genotype** | **Exp.** | ***n*** | **Reprod. adult** | **Mild delay** | **Severe delay** |
| --- | --- | --- | --- | --- | --- | --- |
| ZM7963 | *hpDf761*II;*daf-28(tm2308)*V. | 1 | 10 | 0 | 0 | 10 |
|  |  | 2 | 26 | 0 | 0 | 26 |
| TGF141 | *drxEx21*[p*daf-28*::*daf-28*::mCherry] (high); *hpDf761*II;*daf-28(tm2308)*V. | 1 | 39 | 16 | 19 | 4 |
|  |  | 2 | 107 | 54 | 30 | 23 |
|  |  | 3 | 100 | 32 | 34 | 34 |
|  |  | 4 | 102 | 41 | 30 | 31 |
| TGF141 | Non-Tg sibling | 1 | 22 | 0 | 0 | 22 |
|  |  | 2 | 45 | 0 | 0 | 45 |
|  |  | 3 | 8 | 0 | 0 | 8 |
|  |  | 4 | 21 | 0 | 0 | 21 |
| TGF141 | *drxEx21*[p*daf-28*::*daf-28*::mCherry] (low); *hpDf761*II;*daf-28(tm2308)*V. | 1 | 52 | 7 | 8 | 37 |
| TGF162 | *drxEx21*[p*daf-28*::*daf-28*::mCherry] (high); | 1 | 98 | 49 | 33 | 16 |
|  | *daf-28(sa191)*V. | 2 | 112 | 93 | 8 | 11 |
|  |  | 3 | 102 | 63 | 12 | 27 |
| TGF162 | Non-Tg sibling | 1 | 13 | 2 | 3 | 8 |
|  |  | 2 | 28 | 12 | 3 | 13 |
|  |  | 3 | 38 | 14 | 4 | 20 |

| **Strain** | **Genotype** | **Exp.** | ***n*** | **Reprod. adult** | **Mild delay** | **Severe delay** |
| --- | --- | --- | --- | --- | --- | --- |
| TGF073 | *daf-7(e1372)*III. | 1 | 62 | 1 | 57 | 4 |
|  |  | 2 | 71 | 0 | 61 | 10 |
|  |  | 3 | 59 | 0 | 53 | 6 |
| TGF163 | *drxEx20*[p*daf-7*::mCherry::*daf-7*]; | 1 | 84 | 83 | 1 | 0 |
|  | *daf-7(e1372)*III. | 2 | 126 | 122 | 4 | 0 |
|  |  | 3 | 96 | 92 | 4 | 0 |
|  |  | 4 | 102 | 93 | 9 | 0 |
|  |  | 5 | 126 | 72 | 49 | 5 |
| TGF163 | Non-Tg sibling, First generation | 1 | 17 | 13 | 3 | 1 |
|  |  | 2 | 38 | 35 | 3 | 0 |
|  |  | 3 | 28 | 24 | 4 | 0 |
|  |  | 4 | 30 | 28 | 2 | 0 |
|  |  | 5 | 27 | 3 | 13 | 11 |

| **Strain** | **Genotype** | **Exp.** | ***n*** | **Reprod. adult** | **Mild delay** | **Severe delay** |
| --- | --- | --- | --- | --- | --- | --- |
| TGF154 | *daf-28(sa191)*V;*cuIs5* "Dim" | 1 | 54 | 0 | 49 | 5 |
|  |  | 2 | 65 | 7 | 42 | 16 |
|  |  | 3 | 116 | 0 | 73 | 43 |
| TGF154 | *daf-28(sa191)*V;*cuIs5* "Bright" | 1 | 122 | 12 | 109 | 1 |
|  |  | 2 | 62 | 26 | 32 | 4 |
|  |  | 3 | 124 | 2 | 96 | 26 |

| **Strain** | **Genotype** | **Exp.** | ***n*** | **Reprod. adult** | **Mild delay** | **Severe delay** |
| --- | --- | --- | --- | --- | --- | --- |
| TGF164 | *drxEx20*[p*daf-7*::mCherry::*daf-7*]; | 1 | 94 | 73 | 18 | 3 |
|  | *daf-28(sa191)*V. | 2 | 117 | 95 | 11 | 11 |
|  |  | 3 | 134 | 115 | 8 | 11 |
|  |  | 4 | 113 | 87 | 20 | 6 |
|  |  | 5 | 107 | 93 | 7 | 7 |
|  |  | 6 | 126 | 95 | 28 | 3 |
|  |  | 7 | 109 | 82 | 25 | 2 |
| TGF164 | Non-Tg sibling, First generation | 1 | 12 | 10 | 1 | 1 |
|  |  | 2 | 11 | 9 | 1 | 1 |
|  |  | 3 | 15 | 11 | 1 | 4 |
|  |  | 4 | 28 | 15 | 4 | 9 |
|  |  | 5 | 35 | 30 | 1 | 4 |
|  |  | 6 | 34 | 22 | 6 | 6 |
|  |  | 7 | 47 | 39 | 1 | 7 |
| TGF164 | Non-Tg sibling, Second generation | 1 | 147 | 8 | 41 | 98 |
| TGF157 | *drxEx20*[p*daf-7*::mCherry::*daf-7*]; | 1 | 102 | 0 | 0 | 102 |
|  | *hpDf761*II;*daf-28(tm2308)*V. | 2 | 112 | 0 | 1 | 111 |
| TGF157 | Non-Tg sibling | 1 | 10 | 0 | 0 | 10 |
|  |  | 2 | 26 | 0 | 0 | 26 |

| **Strain** | **Genotype** | **Exp.** | ***n*** | **Reprod. adult** | **Mild delay** | **Severe delay** |
| --- | --- | --- | --- | --- | --- | --- |
| N2AM | Wild-type | 1 | 13 | 13 | 0 | 0 |
|  |  | 2 | 25 | 23 | 1 | 1 |
| JT191 | *daf-28(sa191)*V. | 1 | 50 | 9 | 26 | 15 |
|  |  | 2 | 61 | 3 | 23 | 35 |
| TGF155 | *adEx2202*[*gpa-4*p::*daf-7*];*daf-28(sa191)*V. | 1 | 101 | 17 | 82 | 2 |
|  |  | 2 | 60 | 10 | 50 | 0 |
| TGF155 | Non-Tg siblings | 1 | 50 | 9 | 26 | 15 |
|  |  | 2 | 61 | 3 | 23 | 35 |
| DA2202 | *adEx2202*[*gpa-4*p::*daf-7*]; *daf-7(e1372)*III | 1 | 87 | 85 | 1 | 1 |
|  |  | 2 | 83 | 79 | 4 | 0 |

| **Strain** | **Genotype** | **Exp.** | ***n*** | **Reprod. adult** | **Mild delay** | **Severe delay** |
| --- | --- | --- | --- | --- | --- | --- |
| VC1099 | *hsp-4(gk514)*II. | 1 | 102 | 101 | 1 | 0 |
|  |  | 2 | 85 | 82 | 3 | 0 |
|  |  | 3 | 125 | 109 | 15 | 1 |
| JT191 | *daf-28(sa191)*V. | 1 | 103 | 19 | 75 | 9 |
|  |  | 2 | 123 | 3 | 75 | 45 |
|  |  | 3 | 118 | 15 | 53 | 50 |
| TGF158 | *uthIs270*[*rab-3*p::*xbp-1*s + *myo-2*p::tdTomato]; | 1 | 99 | 70 | 26 | 3 |
|  | *daf-28(sa191)*V;*zcIs4*. | 2 | 31 | 19 | 10 | 2 |
|  |  | 3 | 135 | 101 | 33 | 1 |
| TGF151 | *hsp-4(gk514)*II;*daf-28(sa191)*V;*syIs78* | 1 | 98 | 14 | 3 | 81 |
|  |  | 2 | 88 | 13 | 8 | 67 |
|  |  | 3 | 105 | 8 | 5 | 92 |
